# Air quality and health implications of exposure to ultrafine particle pollution

**DOI:** 10.1093/cvr/cvag136

**Published:** 2026-07-08

**Authors:** Jos Lelieveld, Pantelis Georgiades, Matthias Kohl, Yafang Cheng, Theodoros Christoudias, Jean Sciare, Mihalis Nicolaou, Constantine Dovrolis, Mohammed Fnais, Markku Kulmala, Andreas Daiber, Omar Hahad, Marin Kuntic, Thomas Münzel, Andrea Pozzer

**Affiliations:** Atmospheric Chemistry Department, Max Planck Institute for Chemistry, Hahn-Meitner-Weg 1, 55128 Mainz, Germany; Climate and Atmosphere Research Centre, The Cyprus Institute, P.O. Box 27457, 1645 Nicosia, Cyprus; Climate and Atmosphere Research Centre, The Cyprus Institute, P.O. Box 27457, 1645 Nicosia, Cyprus; Atmospheric Chemistry Department, Max Planck Institute for Chemistry, Hahn-Meitner-Weg 1, 55128 Mainz, Germany; Aerosol Chemistry Department, Max Planck Institute for Chemistry, Mainz, Germany; Climate and Atmosphere Research Centre, The Cyprus Institute, P.O. Box 27457, 1645 Nicosia, Cyprus; Climate and Atmosphere Research Centre, The Cyprus Institute, P.O. Box 27457, 1645 Nicosia, Cyprus; Computation-based Science and Technology Research Centre, The Cyprus Institute, Nicosia, Cyprus; Computation-based Science and Technology Research Centre, The Cyprus Institute, Nicosia, Cyprus; Department of Geology and Geophysics, King Saud University, Riyadh, Saudi Arabia; Institute for Atmospheric and Earth System Research, University of Helsinki, Helsinki, Finland; Department of Cardiology, University Medical Center of the Johannes Gutenberg University Mainz, Mainz, Germany; German Center for Cardiovascular Research, Partner Site Rhine-Mainz, Mainz, Germany; Department of Cardiology, University Medical Center of the Johannes Gutenberg University Mainz, Mainz, Germany; German Center for Cardiovascular Research, Partner Site Rhine-Mainz, Mainz, Germany; Department of Cardiology, University Medical Center of the Johannes Gutenberg University Mainz, Mainz, Germany; German Center for Cardiovascular Research, Partner Site Rhine-Mainz, Mainz, Germany; Department of Cardiology, University Medical Center of the Johannes Gutenberg University Mainz, Mainz, Germany; German Center for Cardiovascular Research, Partner Site Rhine-Mainz, Mainz, Germany; Atmospheric Chemistry Department, Max Planck Institute for Chemistry, Hahn-Meitner-Weg 1, 55128 Mainz, Germany; Climate and Atmosphere Research Centre, The Cyprus Institute, P.O. Box 27457, 1645 Nicosia, Cyprus

**Keywords:** Ultrafine particles, Urban exposure, Air quality, Hazard ratio, Excess mortality

## Abstract

**Aims:**

Ultrafine particles (UFPs) smaller than 100 nm are ubiquitous in polluted air. Although they carry little mass, UFPs have a large exposure surface area, can bypass respiratory defences, translocate into the brain and bloodstream, and impact the heart and other organs. Here, we assess the role of UFPs in air quality and the health burden they impose.

**Methods and results:**

We integrated Earth observations with machine learning to estimate long-term UFP exposure at 1 km resolution, demonstrating that they pose a major air quality concern in urban environments, with annual mean concentrations typically ranging between 10 000 and 30 000 particles/cm^−3^. Model calculations suggest that black and organic carbon are primary components of pollution UFPs. Based on a meta-analysis of epidemiological cohort studies in Europe and North America, we derived a pooled hazard ratio for mortality and combined it with our UFP data. We estimate a mortality density of 35.7 (15.8–65.5) per 100 000 people annually in Europe, and 27.4 (12.9–47.4) per 100 000 in North America, the latter being close to the global mean. We find that UFP exposure and mortality densities are particularly high in South and Eastern Europe. Since observational data for other regions are limited, global calculations primarily depend on modelling. We indicatively estimate that 1.99 (0.81–3.89) million excess deaths per year are attributable to UFP exposure. This could account for approximately 5% of total mortality from non-communicable diseases, to a large degree (about half) due to cardiovascular conditions. Globally, about 91% of UFP-related excess mortality occurs in urban and suburban environments, and much of that (78%) in densely populated urban areas.

**Conclusion:**

Health-improving interventions should target combustion sources in cities, particularly those related to energy consumption, industry, and traffic. An annual air quality limit of 5000 cm^−3^ could reduce global excess mortality by about 45%.

## Graphical Abstract

**Graphical Abstract:**
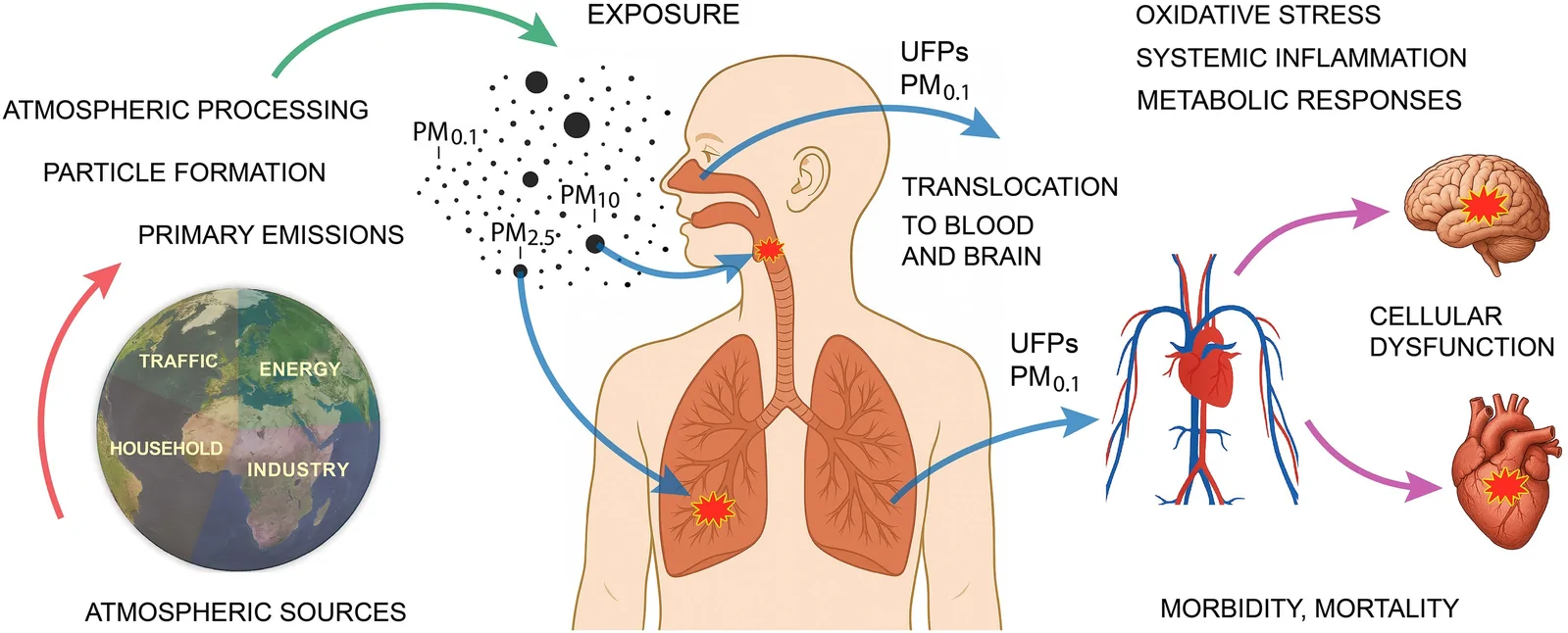

**Time of primary review: 73 days**

**See the editorial comment for this article ‘Ultrafine particles: rethinking air pollution risk’, by M. Rajzer and W. Wojciechowska, https://doi.org/10.1093/cvr/cvag161.**

## 1. Introduction

Long-term exposure to ultrafine particles (UFPs, with diameters less than 100 nm) in polluted air has been linked to adverse health outcomes and increased mortality.^1–5^ Air quality directives address the *mass* concentration of particulate matter with a diameter less than 2.5 µm (PM_2.5_); however, UFPs carry little mass but typically dominate the particle *number* concentration and provide a large surface area that comes into contact with biological tissue upon inhalation.^1,6–8^ UFPs have a high pulmonary retention capacity and induce heterogeneous chemical reactions and harmful biological activity.^9–12^ While PM_2.5_ affects the bronchi and lungs, UFPs can deposit in the alveolar region and translocate into the blood and brain through respiratory and olfactory routes.^13^ This unique biodistribution is particularly relevant for cardiovascular health. Mechanistic studies demonstrate that UFPs induce systemic oxidative stress, endothelial dysfunction, and early atherogenesis—potentially with greater potency than the larger PM_2.5_ fraction.^14–16^

Epidemiological studies have long established PM_2.5_ as a global driver of cardiovascular morbidity and mortality across socioeconomic settings.^17–20^ However, the specific burden attributable to UFPs has remained largely unexplored due to a lack of global exposure data, limited monitoring infrastructure, and poorly characterized emission sources. Addressing these gaps is critical, given the increasing recognition that cardiovascular risk persists even at very low PM_2.5_ concentrations, where UFPs may play a disproportionate role. To elucidate the cardiovascular relevance of UFPs, we adopted a cross-disciplinary approach integrating atmospheric, public health and data science. We generated global UFP exposure estimates using machine learning (ML) fused with satellite and land-use data, with conformal prediction to quantify uncertainties.^21^ We further applied Shapley additive explanations (SHAP) to identify key environmental and anthropogenic drivers of UFP concentrations.

To quantify health impacts, we conducted a meta-analysis of mortality hazard ratios from large cohort studies in North America and Europe,^22–24^ and combined the risk functions with 1-km UFP fields using established Global Burden of Disease methodology.^25,26^ We used a mechanistic Earth system model^27–29^ to attribute UFP exposure to specific anthropogenic emission sources—including traffic, fossil-fuel energy production, industry, and domestic energy use. This integrated framework provides the first global, data- and model-based analysis of UFP exposure and associated mortality, with direct implications for cardiovascular pathophysiology, prevention, and policy.

## 2. Methods

### 2.1 High-resolution UFP exposure data

The ML model fuses satellite data, geographical information, and ground-based UFP measurements.^21^ Datasets encompass residential and non-residential built-up areas, urban, industrial, and commercial complexes, road networks, NO_2_ and PM_2.5_ measurements, air pollution emission inventories, e.g. of CO, CO_2_, NO_x_ (NO + NO_2_), black carbon (BC), population density and meteorological re-analyses. The UFP measurements were collected across continents, though the data availability is mainly limited to North America and Europe. Data partially describe total particle number concentrations (PNCs) based on long-term (>150 days) data from 155 locations. Since Earth observation data have varying resolutions, all predictors and targets were regridded and downscaled onto a common 1 km mesh. The NO_2_ dataset’s grid served as a reference, with variables natively available at 1 km (NO_2_, PM_2.5_, Global Human Settlement Layer datasets, and population) directly mapped, land-use fractions computed within each 1 km cell, and coarser emission inventories redistributed to the underlying 1 km cells using weights proportional to built-up volume and population density. The final dataset spans the period 2010 to 2019 and was used to derive the mean PNCs over land for each year at 1 km global resolution (see Supplementary Materials).

To first derive the PNCs, we employed the Extreme Gradient Boosting (XGBoost) algorithm, using annual-mean predictors on the 1 km NO_2_ grid. We established the optimal set of hyperparameters via grid search over the parameter space and cross-validation (number of trees = 250, maximum depth = 10, learning rate = 0.03, subsample = 0.75, colsample_bytree = 0.75). We used conformal prediction (bootstrap and jacknife+) to determine uncertainties quantitatively. Evaluation of the results, i.e. of the gridded PNCs against 10% test data (randomly allocated), indicates a mean relative error of 29–35% in urban and suburban areas and spatial and temporal correlation coefficients (*r*^2^) of 0.77 or higher. PNCs are often used as a proxy for UFPs. Since approximately 40% of the available PNC measurement data included size information, we tested this proxy assumption by estimating the fraction of UFPs. Results confirm that UFPs dominate PNCs, contributing about 91% on average (hence, ∼9% of the PNC data represent particles >100 nm). The data’s probability distribution demonstrates that the UFP fractions of PNCs are tightly clustered around the mean with low variance, indicating robust, consistent results. From this, a bounded confidence interval (Beta-distribution–based percentile interval) of the UFP fraction of 0.91 ± 0.12 was derived (see Supplementary Materials).

The SHAP method was employed to quantify the contributions of specific environmental and meteorological factors used as ML input features, which complements the conformal predictions. The resulting ranking indicates a leading influence of built-up areas, NO_2_ concentrations, BC emissions, PM_2.5_ concentrations and, in decreasing order, CO emissions, human settlement data, the boundary layer height, population density, CO_2_ and NO_x_ emissions, and finally, temperature and precipitation. The SHAP results indicate that anthropogenic UFP emissions in densely populated urban areas dominate exposure. They also implicate fossil fuel combustion, given the close relationship with leading features such as NO_2_ concentrations and BC emissions.

### 2.2 Earth system model

We have employed a data-constrained Earth system model to compute the exposure to particle concentrations and attribute them to source categories. The model simulating atmospheric chemistry and climate (EMAC) used in this study was applied at T63 resolution (about 1.87° latitude and longitude, 180–190 km), with 31 hybrid terrain-following pressure levels up to 10 hPa (∼30 km altitude).^27–29^ The model applies Newtonian relaxation (‘nudging’) towards meteorological reanalysis data from the European Centre for Medium-Range Weather Forecasts for 2015, which we assume is representative of the long-term population exposure to air pollution. This year was also selected because available emission inventories are consistent with annual UFP measurement data for this period, and we did not detect significant UFP trends and only minor interannual variability in our ML-derived dataset for the period 2010–2019. The anthropogenic emissions of trace gases and particles used as model input have been adopted from the Community Emission Data System (CEDS).^30^

The EMAC model simulates gas-phase and heterogeneous atmospheric chemistry using coupled sub-models that represent photochemical oxidation of natural and anthropogenic emissions, including detailed volatile organic compound chemistry.^31^ Organic aerosol evolution is treated with a volatility-based scheme linking gas and particle phases through thermodynamic partitioning, and extending to inorganic compounds and water uptake.^32^ Aerosol microphysics is modelled with seven interacting lognormal size modes covering nucleation to coarse particles, allowing condensational growth, evaporation, and coagulation.^33^ New particles form via multiple nucleation pathways, including ion-induced processes.^34^ Particle removal occurs through dry deposition, sedimentation, and wet scavenging.^35^ The model performance has been extensively validated against ground-based and satellite measurements.^28,29^

Since the relatively coarse grid resolution of the EMAC model does not accurately capture concentration gradients near strong sources of UFPs, we downscaled the simulation results in two steps: First from 1.87° to 0.5°, and then to 0.1° latitude and longitude (about 10 km by 8 km at mid-latitudes), using available emission inventories (see Supplementary Material).

### 2.3 Estimating the mortality burden

Excess mortality attributable to the long-term exposure to UFP (or attributable mortality) at geographical co-ordinates *x* and *y*, [*M* (*x,y*)], has been calculated by:


(eq. 1) $$M(x,y)=\underset{j}{\sum }\;AF_{j}[X(x,y)]\cdot B_{j}(x,y)\cdot P(x,y)$$

where *j* refers to the age category, here referring to the adult population of 25 years and older, and *X* is the concentration of UFPs. Excess mortality is a metric that measures the number of deaths attributed to UFP during a defined period (in this case, one year), i.e. those that would not have occurred in the absence of exposure.^36^ The *AF* is the attributable fraction of the baseline mortality *B*, and *P* is the population at the geographical co-ordinates, i.e. the 1 km resolution grid cells for which we computed UFP exposure. The baseline mortality, an indicator for a society's overall health, living conditions and organization, and the population data have been adopted from the GBD.^25,26^ The *AF* has been derived from the hazard ratio (*HR*) associated with all-cause (non-accidental) mortality, assuming it represents the relative risk (*RR*) associated with the exposure to UFP from cohort studies in the USA, Canada, and the Netherlands, representing in total 13.3 million participants:


(eq. 2) $$AF_{j}=(RR_{j}(\mathrm{UFP})-1)/RR_{j}(\mathrm{UFP})$$

A log-linear exposure response function (ERF) was applied to describe the dependency of *RR* on the long-term mean concentration of UFPs, according to *RR_j_* = *exp* (β × *X*). The factor β was estimated using a meta-analytic approach, pooling *HRs* using a random-effects model, which assigns greater weight to studies with more precise estimates to improve the precision of the pooled estimate. The log-linear *RR* function is widely used to estimate health effects from PM_2.5_ and other pollutants, including in the GBD. Since β is a small positive number, the shape of the curve is nearly linear. Since PM_2.5_ concentrations can range over several orders of magnitude, integrated exposure–response (IER) and other functions have been introduced to flatten the curve at very high concentrations, thereby preventing unrealistically high results. Since long-term mean UPF concentrations may vary less dramatically, we expect such effects to be less significant. Nevertheless, this has not been investigated, and overestimates of UFP-related mortality at the high end of exposure cannot be excluded. Therefore, these estimates should be considered as an upper bound until high-exposure long-term studies become available.

The mortality calculations consider counterfactual levels assuming 285 cm^−3^ and 1000 cm^−3^ as theoretical minimum risk exposure levels (TMREL). The former is the lowest level in our UFP dataset worldwide over land, and the latter represents typical clean background levels in remote rural areas. We used a TMREL of 285 cm^−3^ in our main results, and a TMREL of 1000 cm^−3^ in a sensitivity analysis. This counterfactual assumption could affect the results by overestimating mortality at the lower end of the exposure range. Similar arguments apply to PM_2.5_, as mortality can increase rapidly at very low exposure levels.^37^ Global disease burden analyses often employ a PM_2.5_ counterfactual range below the WHO guideline concentration of 5 µg/m^3^.^25,26^ We find that using 1000 cm^−3^ instead of 285 cm^−3^ reduces the global excess mortality from UFP exposure by 8.6%. The 95% CIs were derived by incorporating uncertainties in the *RR* function, mortality data, and UFP exposure, yielding an overall 95% CI of about a factor of two.

## 3. Results

### 3.1 Meta-analysis of hazard ratios

We estimated *RR* and the attributable fraction (*AF*) in eq. 2, using a meta-analytic approach, pooling *HR*s with a random-effects model weighted by inverse variance. Studies with smaller standard errors and confidence intervals (CIs) contribute greater weight to the pooled estimate, while accounting for both within-study variance and between-study heterogeneity. In the Supplementary Materials, we provide meta-analytical diagnostics and test different methods, showing that the pooled *HR* estimate is robust and differs only in its CIs (Supplementary material online, Figure  *S6*). Supplementary material online, Figure  *S6* also shows all *HRs* expressed per 10 000 cm^−3^ UFP exposure increment, converted from the different cohort study results. Supplementary material online, Figure  *S7* shows the *AF* curves derived, used to calculate all-cause excess mortality. We reiterate that, in most cohort studies, PNC is used as a proxy for UFP exposure, supported by our estimate that UFPs contribute approximately 91% of total PNC on average.

We used *HRs* for UFP exposure and total particle number concentrations from recent studies in North America and Europe. Unfortunately, we could not use the earlier study by Ostro *et al*.^4^ who reported *HR*s based on UFP mass concentrations rather than number concentrations and did not find significant associations with all-cause mortality (but did for mortality from ischaemic heart disease). Mass and number concentrations of UFPs cannot be directly compared, and the study used 4 km-resolution data, which may have lost some information relative to 1 km-resolution data. Nevertheless, Ostro *et al*. reported that, across all constituents investigated, the *HR* model fit for ischaemic heart disease mortality was better for UFPs than PM_2.5_.

For all-cause mortality, two recent cohort studies in the USA (618 000 and 396 000 participants) reported *HRs* of 1.03 (95% CI 1.02–1.04) and 1.02 (95% CI 1.00–1.04) per interquartile range (IQR) (∼5000 particles/cm^3^) increment.^22^ The studies applied 1 km-resolution PNC data, assumed representative of UFPs.^38^ The data were spatially aggregated to adjust to residential information available at the census tract level, encompassing several thousand people. Pond *et al*.^22^ reported a significant association with all-cause mortality by applying single-pollutant models. UFP-associated mortality was generally robust to controlling for PM_10_ minus PM_2.5_ (i.e. the relatively coarse size fraction of fine aerosol particles) and SO_2_, but not PM_2.5_. This may be expected as the PM_2.5_ and UFP size fractions overlap, whereas the other metrics are independent. Furthermore, aggregating the UFP data to a coarser resolution may have removed some fine-scale features, as in Ostro et al.^4^

The large cohort study in the Netherlands (10.8 million participants) reported an *HR* of 1.012 (95% CI 1.010–1.015 per IQR (∼2700 particles/cm^3^) increment for natural (all-cause) mortality.^23^ This study also applied long-term PNCs, assumed representative of exposure to UFPs.^39^ In agreement with our ML analysis, high correlations between UFPs, NO_2_ and BC were reported. The study found positive associations of UFP exposure at very high spatial resolution with all-cause, cardiovascular disease, (non-malignant) respiratory disease, and lung cancer mortality. In two-pollutant models with NO_2_, PM_10_, and PM_2.5_, the associations for all-cause and lung cancer mortality remained significant, whereas those with cardiovascular and respiratory mortality declined. Bouma *et al*.^23^ mention that their results agree closely with those of Pond *et al*.^22^

The Canadian study (1.544 million adult participants in two large cities) reported an *HR* of 1.073 (95% CI 1.061–1.085) per 10 000 particles/cm^3^ increment.^24^ Significant associations of nonaccidental (all-cause) and disease-specific mortality with UFPs were found, shown to be independent of PM_2.5_, O_3,_ and NO_2_ based on different exposure models. The study was the first to consider possible confounding by particle size, indicating that UFPs are independently associated with mortality in urban environments. Lloyd *et al*.^24^ suggest that previous studies that only applied PNCs may have underestimated (or missed) the health risks. Interestingly, the study found that the largest UFP size fraction was most strongly associated with mortality. This points to particles directly emitted from combustion sources, as the smallest size fraction is more likely from new particle formation. This is consistent with Lloyd et al.’s finding that concentration-response curves levelled off when influenced by a very high number concentration of freshly nucleated particles.^24^

Overall, the outcomes of the four cohort studies agree well after adjustment to the same concentration scale, although the Canadian *HRs* are slightly higher. Lloyd *et al*.^24^ reported that their results were consistent and of similar magnitude when UFP size was excluded. By performing a meta-analysis of these study results, the pooled *HR* for all-cause mortality is 1.057 (95% CI 1.039–1.075) per 10 000 particles/cm^3^ increment. Additional evidence for health risks and mortality was presented in a meta-analysis of long-term exposure to traffic-related air pollution and non-accidental mortality, reporting a significant relationship with BC (reported as elemental carbon), with an *HR* of 1.02 (95% CI 1.02–1.04) per µg/m^3^,^20^ corroborated by a recent Health Effects Institute report.^40^ Since this *HR* is based on particle mass rather than number concentration, the results cannot be directly compared, but considering that a significant fraction of BC is emitted in the UFP size range, it supports the results from the cohort studies applied here.

### 3.2 Exposure to UFPs: North America and Europe

**Figure 1:**
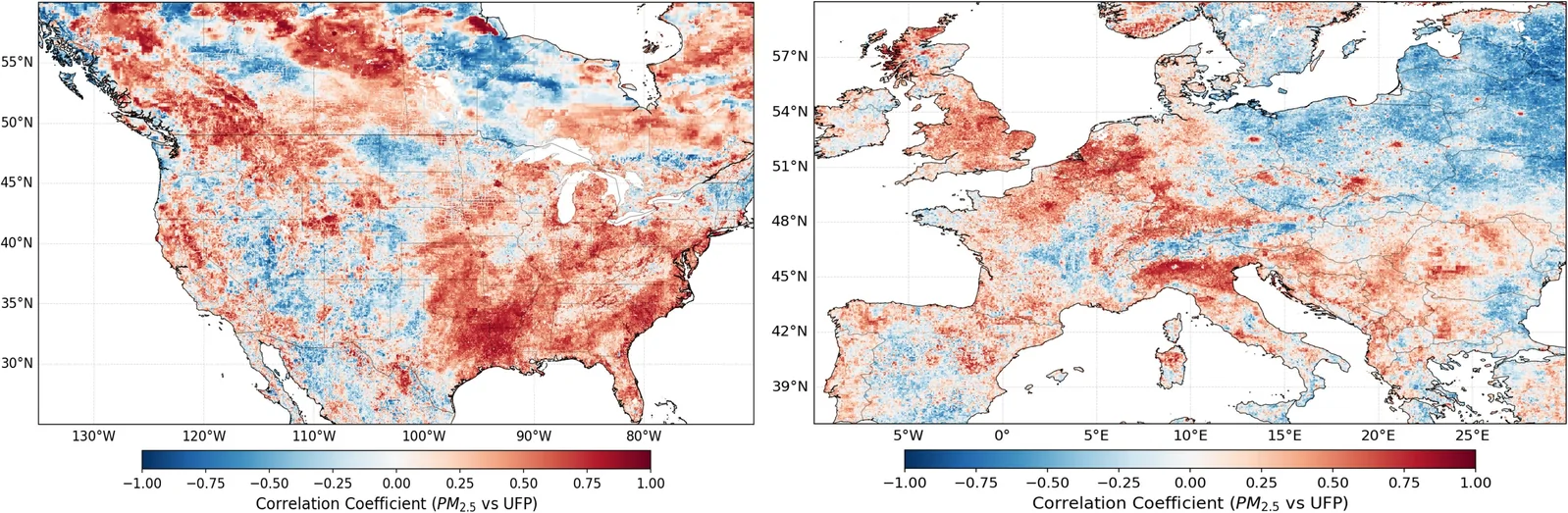
Correlations between PM_2.5_ and UFP concentrations. Correlations in urban environments are typically positive but sometimes low, while in rural areas, they can be positive, low, or negative.

The concentration of PM_2.5_ was identified as the fourth-most-important SHAP feature for predicting UFP concentrations. In *Figure 1*, we show the correlations between our UFP data from 2010 to 2019 and those of PM_2.5_, adopted from Van Donkelaar *et al*.^41^ and available at the same spatial resolution and timeframe. In this section, we focus on North America (25°–60°N, 70°–135°W, ∼360 million inhabitants) and Europe (37–60°N, 10°W–30°E, ∼590 million) as our UFP data predominantly represent these two continents due to the abundance of ground-based measurements. When anthropogenic PM_2.5_ levels are high, UFPs can also be plentiful because of substantial emissions across the entire particle size spectrum. Nonetheless, *Figure 1* shows that their concentrations can both correlate and anti-correlate.

These varying correlations illustrate that PM_2.5_ and UFP sources do not necessarily coincide and might not respond similarly to measures aimed at reducing air pollution. Geographic differences relate to the quantities of primary (directly emitted) and secondary (formed in the atmosphere) aerosol particles, which vary considerably among UFPs and PM_2.5_. Furthermore, UFPs are measured by number, while PM_2.5_ is measured by mass concentration. Our Earth system modelling indicates that pollution UFPs primarily originate from combustion processes. They are mainly composed of primary black and organic carbon particles, along with a substantial fraction of secondary organic compounds (see Supplementary material online, Figure  *S10*). PM_2.5_ contains these species (including UFPs) as well as a large fraction of secondary inorganic components (e.g. sulphate, ammonium, nitrate) and mineral dust. We find that in North America and Europe, and generally in high-income countries, UFP exposure is mainly associated with industrial emissions (39%), fossil energy production (33%), road traffic (20%), and domestic energy use (8%). While PM_2.5_ is significantly influenced by natural and agricultural emissions, this is much less the case for UFPs.

**Figure 2:**
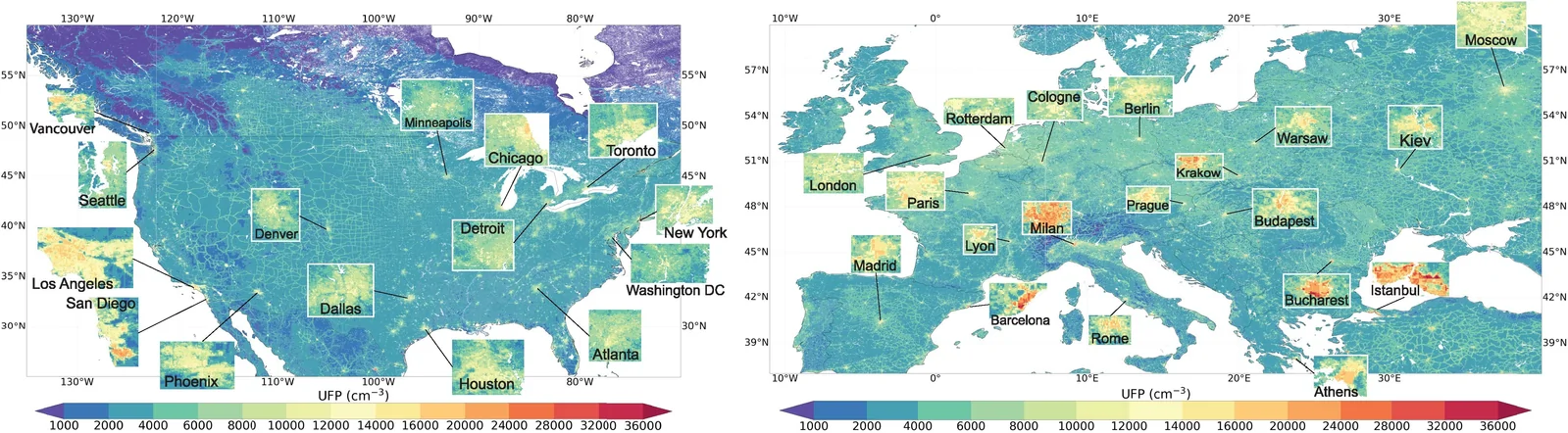
Annual mean UFP number concentrations in particles per cm^3^. Inset panels show expanded views of 34 metropolitan areas.

*Figure 2* displays ML-model-derived annual mean UFP concentrations across North America and Europe, highlighting metropolitan areas with source hotspots, including major traffic routes. High-resolution versions of these images are available in the Supplementary Material. To differentiate suburban and urban areas from rural regions, we used population thresholds of 250 and 800 inhabitants per km^2^, respectively. We find that annual UFP concentrations for 2010–2019 range from 2000 to 3000 cm^−3^ in rural settings up to 40 000 cm^−3^ in highly polluted areas, mainly urban environments. The World Health Organization (WHO) considers 24-hour and 1-hourly mean concentrations of 10 000 cm^−3^ and 20 000 cm^−3^, respectively, as ‘high’, while annual level indicators are not provided.^42^

### 3.3 Global exposure to UFPs

**Figure 3:**
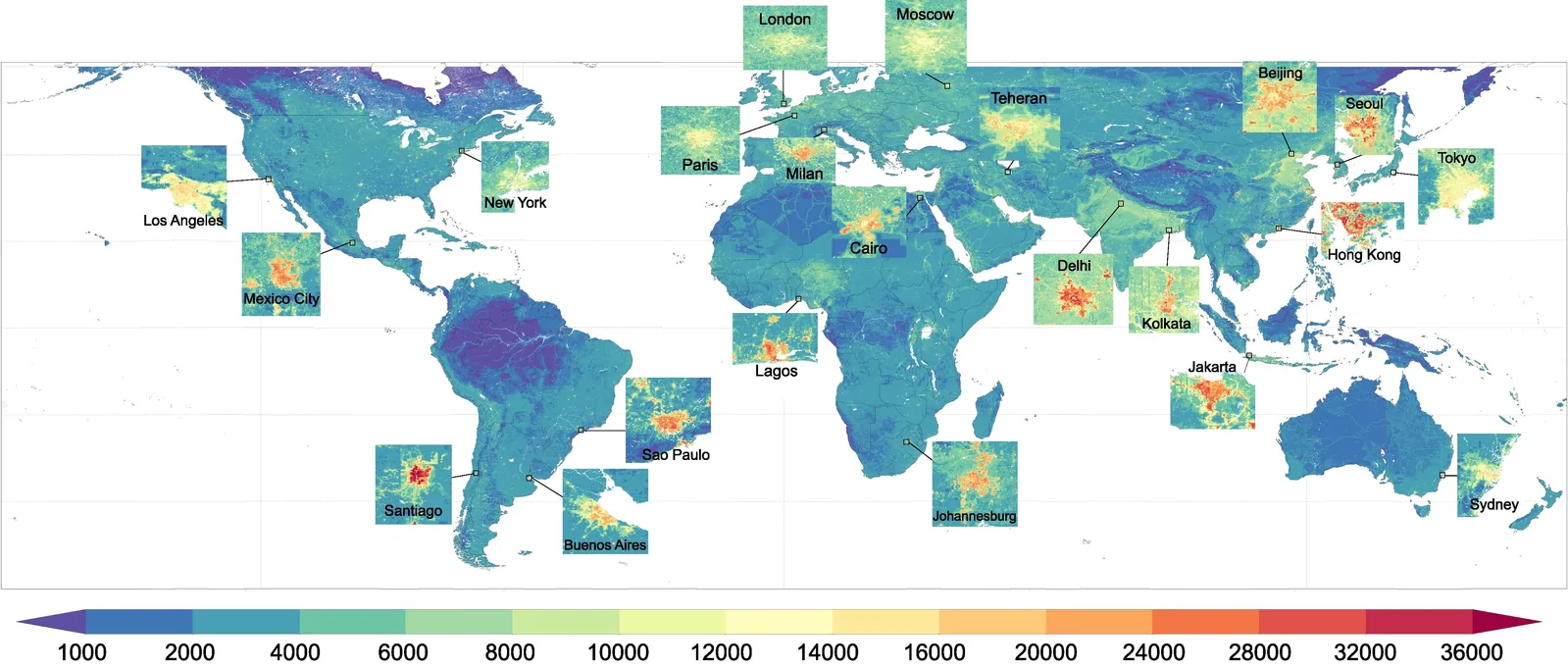
Annual mean UFP distribution. The inset panels show UFP number concentrations (particles per cm^3^) in 22 metropolitan areas, home to about 260 million people.

Since the UFP data coverage outside North America and Europe is limited, we assessed the performance of our ML model using multiple validation strategies.^21^ The conventional train–test split evaluation achieved a correlation coefficient (*r*^2^) of 0.90, whereas a 10-fold cross-validation yielded slightly improved results, with an *r*^2^ of 0.91. However, spatial cross-validation yielded lower performance (*r*^2^ = 0.77). Urban areas exhibited the lowest uncertainties, benefiting from comparatively dense monitoring coverage, whereas rural regions show higher percentage errors—though these are less critical for exposure assessment due to their lower population densities and UFP levels. On the other hand, temporal cross-validation yielded stronger predictive performance with an *r*^2^ of 0.87. This comparatively higher temporal accuracy indicates that the ML model captures interannual variations more effectively than it does spatial patterns. Keeping in mind the reduced ML model performance in data-scarce areas, *Figure 3* presents the global exposure map. A high-resolution version of this image is provided in the Supplementary Materials.

### 3.4 Health burden: North America and Europe

**Figure 4:**
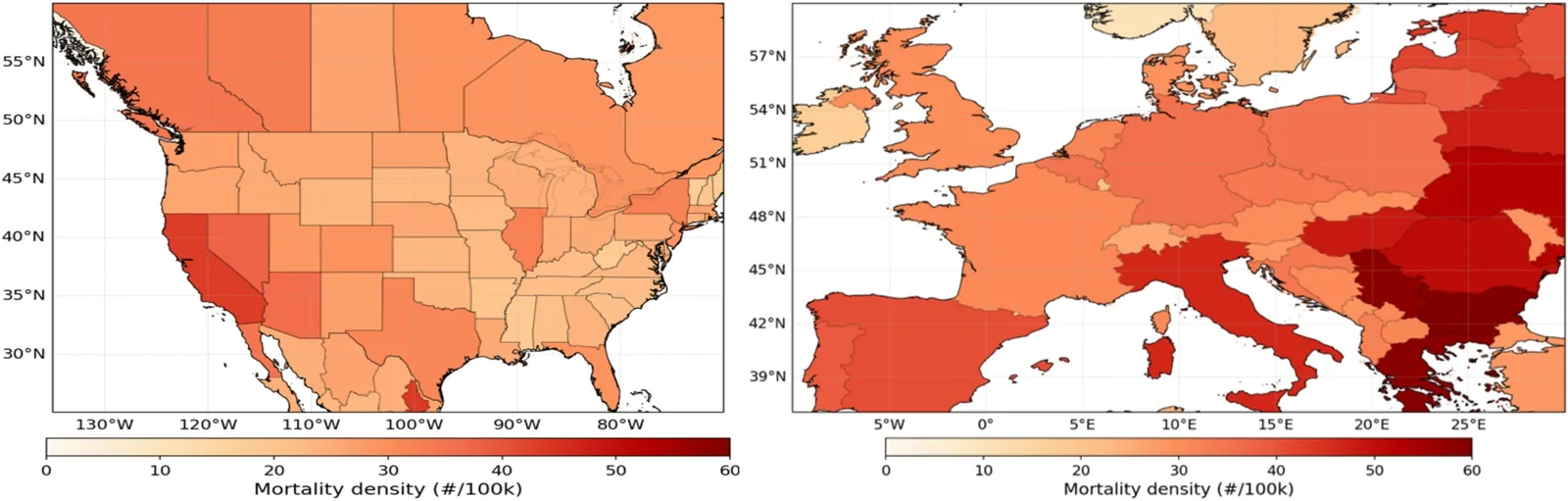
Annual mortality densities. The state-, provincial-, and country-average values are presented per 100 000 population.

Our mortality burden calculations indicate that the annual number of excess deaths due to UFP exposure in North America amounts to 99 540 (95% CI 46 930–171 960), and in Europe 209 840 (95% CI 92 900–384 880). The total number of deaths from non-communicable diseases in these regions is around 8 million annually,^25^ suggesting that nearly 4% are attributable to UFP exposure. The annual mortality densities due to UFPs are shown in *Figure 4*, with an average of 35.7 (95% CI 15.8–65.5) per 100 000 population in Europe and 27.4 (95% CI 12.9–47.4) per 100 000 in North America. Particularly high mortality densities occur in southeastern Europe, such as Greece, with 57.0 (95% CI 28.0–96.9) per 100 000. Eastern and Southern European countries generally have somewhat lower mortality densities than those in the Southeast, but they are still very high. In North America, California stands out with 42.1 (95% CI 22.4–67.7) per 100 000 per year.

By applying a population density threshold of 250 km^−2^, we find that in North America and Europe, 88% of UFP-related excess mortality occurs in urban and suburban areas, with a large part (75%) in densely populated urban areas (>800 km^−2^), indicating that interventions in cities and especially in city centres can contribute effectively to health improvement. Furthermore, we performed sensitivity analyses to assess the reductions in mortality achievable through regulatory interventions. As an illustration, we assumed two UFP concentration benchmarks, 10 000 cm^−3^ (the WHO 24-hour high level) and 5000 cm^−3^. Implementing the 10 000 cm^−3^ and 5000 cm^−3^ limits would reduce UFP-associated mortality in Europe and North America by about 13 and 30%, respectively. A target of 5000 cm^−3^ could be an interim goal to reduce current UFP exposure impacts in these regions by 30%. Note that these percentages are not sensitive to uncertainties in mortality calculations; they remain the same whether mortality is higher or lower. Ultimately, stricter mitigation will be needed, aiming for annual levels well below 5000 cm^−3^.

### 3.5 Global health burden

We reiterate that our global data carries greater uncertainty than data for North America and Europe. While the ML model’s conformal prediction confidence intervals adaptively broaden in areas with low training data density, as considered in the mortality calculations, the *HRs* have been derived in high-income countries rather than in global contexts, so their representativeness for the global South remains uncertain. Given this constraint, and considering the promising agreement between our ML results, Earth system model outcomes, and measurement data, we also conducted a global analysis to provide context for our regional results in North America and Europe. Therefore, the global estimates are indicative and should be interpreted with caution until cohort evidence is more geographically representative.

**Figure 5:**
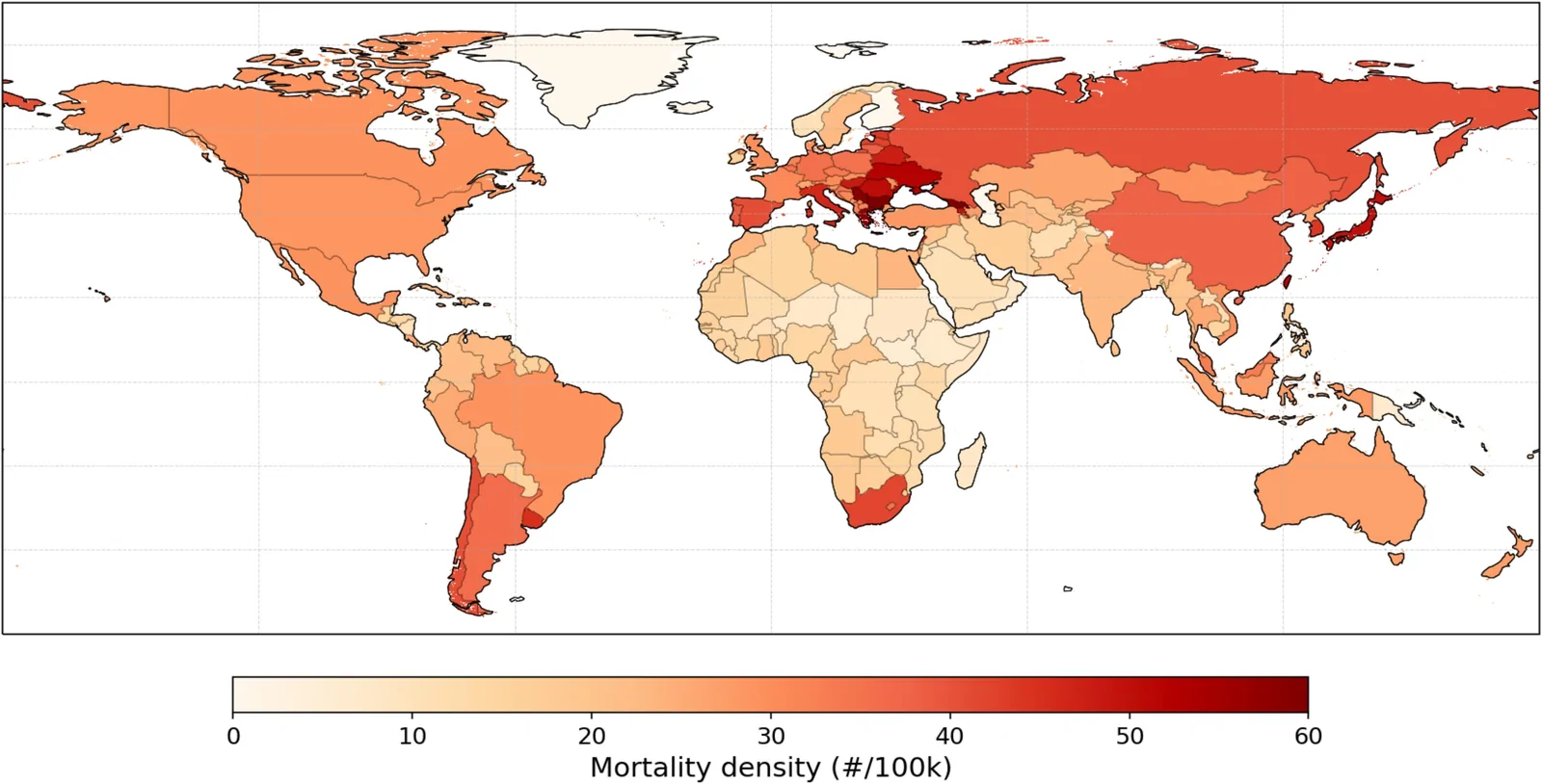
Annual country-average mortality density per 100 000 population.

According to the GBD database,^25^ the total global mortality from non-communicable diseases is 37.1 (95% CI 34.8–39.6) million per year. We estimate that 1.99 (95% CI 0.81–3.89) million excess deaths per year are attributable to UFP exposure, representing approximately 5% of total mortality from non-communicable diseases. The global mean excess mortality density due to UFPs is 28.9 (95%CI 10.6–50.6) per 100 000 population per year is shown in *Figure 5*. The mortality density is relatively low in Sub-Saharan Africa, e.g. 6.8 per 100 000/yr in Chad (where communicable diseases dominate mortality), and relatively high in high-income countries, with about 33.5 per 100 000/yr. Excess mortality densities are relatively high in South and Eastern Europe, South Africa, and East Asia, exceeding 35 per 100 000/yr. The average mortality density in Europe is significantly higher than the global mean. This may not reflect the expectation that emission control measures in the past decades have significantly improved air quality.

By applying the population density threshold of 250 km^−2^, we find that worldwide, 91% of UFP-related excess mortality occurs in urban and suburban environments, and much of that (78%) in densely populated urban areas (>800 km^−2^). For example, in Indonesia, Brazil and Japan, >50 000 yearly excess deaths from UFPs occur in urban areas; in Mexico, this is >30,000, and in Italy, Bangladesh, South Korea and Vietnam, >20,000, suggesting major health benefits from emission controls in cities. A regional comparison indicates that the highest UFP-related urban mortality densities are found in high-income areas of Pacific Asia, Latin America, and some Middle Eastern countries. Notable examples include Singapore, Taiwan, South Korea; Uruguay, Brazil; Israel, Bahrain, and Egypt. We also considered the two UFP concentration limits as potential global intervention targets. Implementing the 10 000 cm^−3^ and the 5000 cm^−3^ benchmarks worldwide could reduce UFP-associated mortality by approximately 22 and 45%, respectively.

### 3.6 Source attribution

To attribute UFP exposure and excess mortality to the pollution sources, emissions in our Earth system model have been aggregated into the following categories: Energy production and industry (from fossil fuel use), domestic (residential and commercial) energy use (mainly wood burning), traffic (fossil fuel use), and the total of minor (other) sources, including agriculture, solvent use, waste burning, and vegetation fires (biomass burning). The model results suggest that exposure to UFPs is predominantly from combustion sources, with up to 75% involving fossil fuels, calculated by combining the traffic, industry and energy sectors.

**Figure 6:**
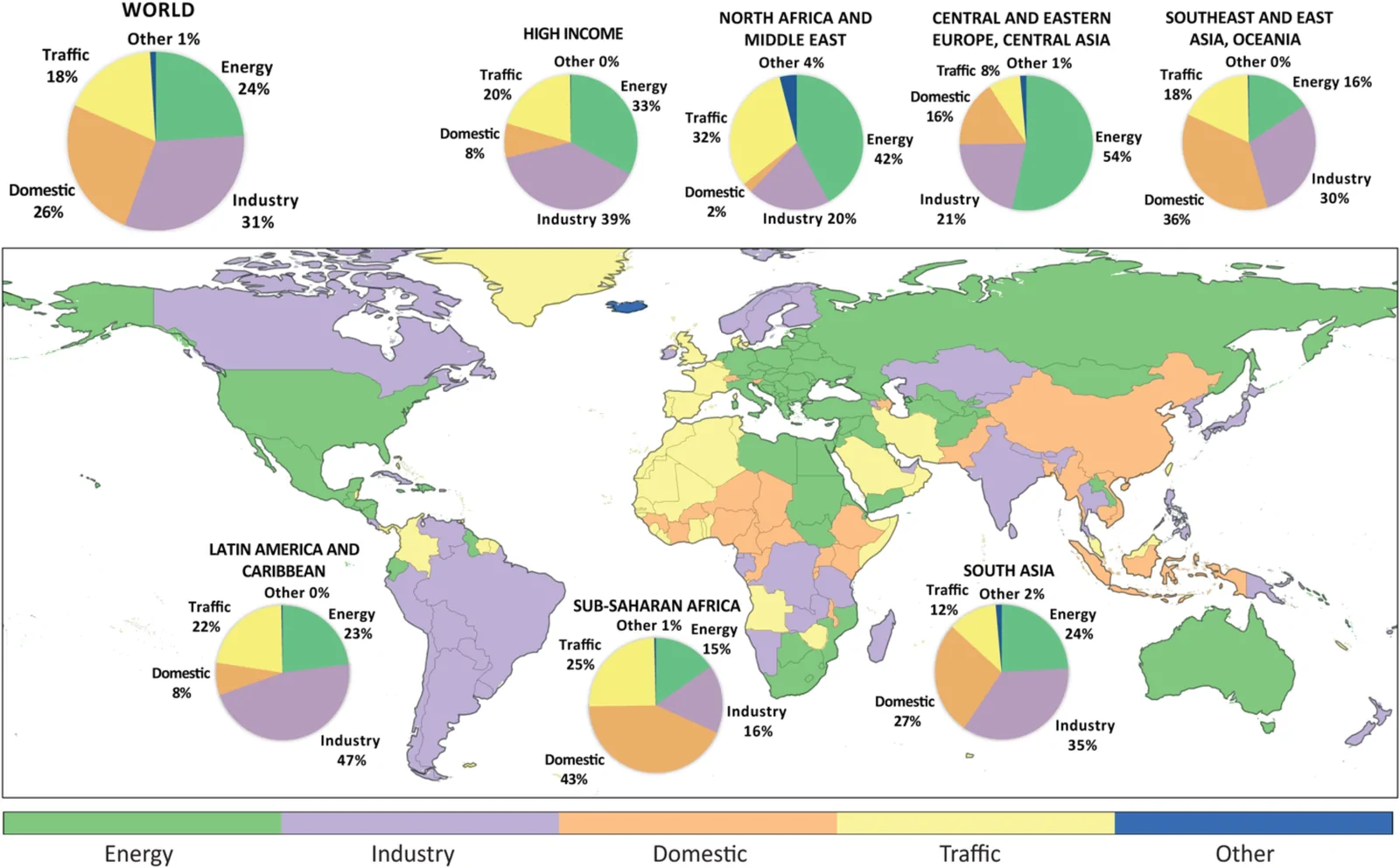
Model-calculated anthropogenic sources of exposure to UFPs. The global map presents the leading sectors per country, and pie diagrams the different sectors for macro-regions. ‘Other’ includes waste treatment, vegetation fires, and agriculture.

*Figure 6* presents the source attribution to different regions and sectors (regions follow the GBD definition; see Supplementary material online, Figure  *S11*). Domestic (residential and commercial) energy applications, such as cooking and heating, mainly rely on solid biofuels (e.g. wood), which significantly contribute to energy use in low- and middle-income countries in South, Southeast, and East Asia and Sub-Saharan Africa. UFP exposure from domestic energy use is less relevant in high-income countries in Europe, the Americas, and East Asia, as well as in North Africa and the Middle East (<8%). In many regions, industry, energy production, and traffic depend on fossil fuels. Industrial applications of fossil fuels include metal production (e.g. iron, steel, aluminium) and the chemical and transformation sectors (e.g. coal and coke production, petroleum refining).

Our Earth system model results show that pollution UFPs are dominated by carbonaceous particles, unlike PM_2.5_, which additionally contains significant fractions of inorganic and mineral compounds. The spatial distributions can differ considerably and partially correlate inversely, especially in non-polluted environments where UFPs depend more strongly on new particle formation. We find that in polluted (primarily urban) environments, exposure is mainly due to primary BC sources and a combination of primary (directly emitted) and secondary (formed in the atmosphere) organic aerosols. Globally, anthropogenic UFP exposure is mainly from industry (31%), followed by domestic energy use (26%), fossil energy production (24%) and road traffic (18%) (*Figure 6*).

## 4. Discussion

### 4.1 Urban exposure and mortality burden

Our high-resolution UFP data reveal steep concentration gradients near sources, particularly in urban environments. We find that 91% of UFP-related excess deaths occur in and around cities, including suburban areas, with 78% in urban centres having populations over 800/km^2^. The mortality burden calculations for North America and Europe (combined) indicate that long-term exposure to UFPs results in 309 380 (95% CI 139 830–556 840) excess deaths annually, translating to an annual mortality density of 32.5 (95% CI 14.7–58.5) per 100 000 population. Recognizing that our global estimates depend on model calculations rather than direct UFP measurements, we estimate 1.99 (95% CI 0.81–3.89) million excess deaths per year. To put this into perspective, global excess mortality from PM_2.5_ has been estimated to range between four and eight million annually, depending on specific exposure-response functions, pollution types and disease categories.^43,44^

Our estimated excess mortality density aligns with a ‘bottom-up’ study that estimated excess deaths from UFP exposure through its effect on cardiovascular disease and related mortality.^13^ That study estimated the annual incidence of cardiovascular disease from UFPs in the European Union at 419 (95% CI 78–712) thousand, resulting in an incidence rate of 93.9 (17.5–159.6) per 100 000 people per year. This rate was significantly higher than the global figure of 76.2 (16.2–115.2) per 100 000 population annually. This appears consistent with the relatively high European mortality density found here, mainly driven by the elevated levels in Southern and Eastern Europe.

### 4.2 Uncertainty and counterfactuals

We have comprehensively accounted for statistical uncertainties in exposure, which adaptively expand in regions with low ML model training data density, yielding an overall 95% CI for mortality estimates within a factor of about 2. This does not include epistemic (knowledge-based) uncertainty. In the Supplementary Materials, we provide a detailed account of data uncertainties. Furthermore, the representativeness of UFP measurements and epidemiological cohort studies in high-income countries for the global South remains largely undetermined. Potential effect modifiers include the baseline CVD risk profile, competing infectious mortality, healthcare access, and source mixture/combustion types, which are particularly relevant for low- and middle-income countries.

Because UFP levels can be highly correlated with other combustion-related pollutants and are typically estimated with considerable exposure uncertainty, multi-pollutant models in cohort studies may yield attenuated mortality estimates and obscure underlying associations. Lloyd et al. (2024) observed possible confounding by particle size, suggesting that studies that do not account for it may underestimate mortality risks associated with UFP exposure. In our meta-analysis, we emphasize single-pollutant models, which provide a stable signal of pollution linked to spatial variation in UFPs while acknowledging that this signal likely reflects the broader combustion-related pollution mixture.

Another limitation concerns the counterfactual level, for which we assumed a TMREL of the lowest observed UFP concentration of 285 cm^−3^. If this level were higher, e.g. 1000 cm^−3^, the predicted mortality burden in North America and Europe would decrease by about 11%, and by about 8–9% for global estimates. Sensitivity to TMREL is greatest in regions with relatively low background exposure; thus, attributable fractions there should be interpreted as more dependent on the counterfactual assumption.

The Supplementary Materials include Supplementary material online, *Tables S1-S6*, which provide results on population-weighted exposure to UFPs, mortality estimates for the two TMREL levels mentioned above, including 95% CIs (indicated as MIN–MAX ranges), percentage contributions from source sectors, and contributions to the variance underlying the 95% CIs for GBD-defined regions and all countries worldwide. We find that the 95% CIs for attributable mortality are substantially driven by baseline mortality rates, which vary significantly across countries due to social determinants of health, exposure uncertainty (from conformal prediction), and exposure response functions (ERFs, from meta-analysis). Globally, uncertainty in baseline mortality, UFP exposure, and ERFs account for about 26%, 41%, and 33%, respectively, of the total 95% CIs.

### 4.3 Disease-specific risks

The recent cohort study by Lloyd *et al*.^24^ differentiated between UFP size and number, concluding that exposure is independently associated with all-cause mortality. They observed that *HRs* are lower for disease-specific and all-cause mortality when size is not distinguished. The *HRs* for all-cause mortality across studies align well for single-pollutant analyses but differ when multi-pollutant models are employed, suggesting that such models may better account for interrelationships between pollutants.^40^ Further support for morbidity and mortality related to UFP exposure is provided by the large number of studies reporting disease-specific risks, such as arterial hypertension, diabetes, cardiovascular disease, and dementia.^20,45–53^

### 4.4 Particle characteristics and sources

There is increasing evidence that the adverse health effects of PM_2.5_ persist even at very low concentrations.^37^ It is plausible that at these levels, UFPs play an important role, as they are unregulated and generally abundant. Our comparison of UFP and PM_2.5_ data shows varying correlations, indicating that emission control measures may not affect both species equally. For instance, catalytic converters and exhaust filters are effective at reducing the mass of fine particulate matter, but not UFP numbers, which may even increase in emissions.^54,55^ Further research should involve air quality networks that include UFPs and/or PNCs in their continuous measurements and studies across different geographic regions to improve global representativeness.

The atmospheric model results indicate that in polluted environments, UFPs mainly consist of black and organic carbon from combustion sources. This is supported by urban measurements and chemical fingerprinting of UFPs in human serum.^56–58^ The particles may also carry trace metals and toxic substances such as low-volatile aromatics; their interaction with biological tissue is facilitated by their large surface-to-mass ratio.^11^ Pollution UFPs contain a small fraction of inorganics and lack mineral compounds, making them distinct from PM_2.5_. Natural UFPs are found to contribute little to exposure and mainly occur in rural settings, which are considered a lower-priority health concern. Natural sources are expected to be generally less hazardous, including those from organic and inorganic compounds that contribute to new particle formation.^59–61^

### 4.5 High UFP levels from new particle formation

Newly formed particles of a few nanometres are effectively transferred in air by Brownian forces and, upon inhalation, primarily deposit in the upper respiratory tract from which clearance is relatively fast; larger UFPs (>10 nm) reach the alveolar region, translocate into circulation, and affect the heart and other organs.^1,13^ Lloyd *et al*.^24^ report decreasing hazard ratios with UFP size and reduced responses at very high number concentrations (>20 000 cm^−3^). This suggests that primary emissions, rather than newly formed particles, which can be abundant through nucleation events, are the main concern. Here, we do not adjust *HR*s at very high UFP concentrations, unlike functions that account for very high PM_2.5_ levels,^43^ as this information is currently unavailable. This could lead to an overestimation of excess mortality at the upper UFP concentration range. Therefore, mortality estimates in the highest-exposure tail should be interpreted as a potential upper bound.

### 4.6 Policy-relevant thresholds, implications for mitigation

Our SHAP analysis and atmospheric model results indicate that anthropogenic exposure to UFPs mainly comes from burning fossil fuels for energy, industry, and transport. Globally, fossil fuel consumption accounts for nearly 75% and over 90% in high-income countries. In low- and middle-income countries, domestic energy sources such as wood burning also contribute significantly (30–40%). This suggests that renewable, non-combustion energy sources can provide notable health benefits. Air pollution policies should aim for annual exposure levels well below the WHO 24-hour ‘high’ threshold of 10 000 cm^−3^. In high-income countries, an annual mean UFP concentration benchmark of 10 000 cm^−3^ would prevent about 13% of excess deaths per year, while a level of 5000 cm^−3^ could prevent 30%. Globally, a benchmark of 5000 cm^−3^ reduces excess mortality by about 45%, which might qualify as an interim air pollution control target. Note that these percentages are not sensitive to uncertainties in mortality calculations. Ultimately, stricter limits will be needed to meet clean air quality standards. Banning combustion sources in cities, including those related to traffic, industry, and energy production, would be an effective intervention to improve public health.

Translational perspective Ultrafine particles (UFPs) are an under-recognized yet highly relevant cardiovascular risk factor. By integrating Earth observations, machine learning, cohort risk estimates, and source attribution, this study maps long-term UFP exposure at 1 km resolution and attributes it to mortality. Annual urban concentrations commonly reach 10 000–30 000 particles/cm^3^, with an indicative two million excess deaths globally, about half from cardiovascular outcomes. Most deaths occur in cities, implicating combustion from traffic, industry, energy, and domestic fuels. The findings make UFPs a clinically actionable target for cardiovascular prevention, urban policy, monitoring networks, and non-combustion clean-air interventions, especially in high-risk cardiometabolic populations and densely populated urban corridors.

## 5. Summary and clinical implications

Ultrafine particles (UFPs, <100 nm) pose a significant cardiovascular risk that remains unregulated. Global data indicate that urban annual concentrations characteristically reach 10 000–30 000 particles/cm^3^. Growing evidence of the harmful health effects of UFPs has led the WHO and the European Union to classify them as ‘contaminants of emerging concern’ for potential inclusion in air quality frameworks. Epidemiological evidence shows that exposure to UFPs is independently associated with increased cardiovascular risk,^45^ including higher hospital admissions and links to conditions such as hypertension, diabetes, heart failure, myocardial infarction, and overall mortality.^22–24,46–48^ The pooled hazard ratio of 1.057 per 10 000 particles/cm^3^ increase, derived here and combined with high-resolution exposure data, suggests approximately two million excess deaths globally each year. The WHO estimates that cardiovascular diseases account for about half of premature deaths from non-communicable diseases. In comparison, 47–67% of excess mortality due to exposure to PM_2.5_ has been attributed to cardiovascular diseases.^62^

Human inhalation experiments and lung modelling show that UFP deposition in the 10–100 nm range exceeds 90%, primarily in the alveolar region.^63,64^ Animal studies further reveal that, unlike PM_2.5_, which predominantly causes pulmonary inflammation, UFPs can translocate into systemic circulation and target extrapulmonary organs, including the brain, heart, and vasculature.^9,13,65^ Controlled exposure experiments in humans and animals demonstrate altered blood pressure regulation, endothelial dysfunction, atherogenesis, impaired coronary microcirculation, myocardial injury, and cognitive dysfunction.^65–68^ Mechanistic evidence through multi-omics profiling and targeted testing also indicates that UFPs induce systemic inflammation, oxidative stress, endothelial dysfunction and coagulation, which are associated with hypertension, arterial stiffness, and increased cardiovascular disease risk.^5,50^

UFPs can furthermore reach the brain via the olfactory bulb and have been associated with neural inflammation, providing a plausible mechanistic link to neurodegenerative conditions such as Alzheimer’s and Parkinson’s disease.^65,66^ They can cross or disrupt the blood–brain barrier, leading to oxidative stress and neuroinflammation.^69–73^ Exposure to traffic-derived UFPs induces oxidative damage and inflammatory responses in olfactory epithelial cells, activating astrocytes and microglia and upregulating neuroinflammatory markers.^71,74^

At the cellular level, targeted delivery studies and biopsies from humans, animals, and cell cultures show that UFPs readily cross plasma membranes and impair mitochondrial function.^75^ Reported effects include disrupted redox homeostasis and mitochondrial ultrastructural damage,^76^ as well as exacerbation of ischaemia–reperfusion injury by modulating mitochondrial permeability transition pore opening.^77^ In healthy volunteers, UFP exposure led to myocardial particle accumulation and increased markers of oxidative stress and cardiovascular risk.^78^ Moreover, chronic tissue stress can propagate systemic metabolic dysregulation through signalling pathways, reshaping the metabolome towards maladaptive states.^79,80^ UFP exposure has been shown to impair mitochondrial ATP production while elevating reactive oxygen species.^81,82^

Collectively, these and our new findings underscore the importance of incorporating UFPs into health risk assessment frameworks and air-quality standards. Their capacity to induce systemic injury highlights their role in cardiovascular disease, supporting the need for targeted public health interventions. Routine UFP monitoring at air quality stations would enable more accurate exposure assessment for long-term epidemiological research, though implementation remains challenging—particularly in low- and middle-income countries where monitoring infrastructure is limited. Expanding measurement networks, combined with our machine learning and Earth system modelling approaches, offers a pathway to address these exposure data gaps.

## Supplementary Material

cvag136_Supplementary_Data

## Data Availability

The global results for PNCs and UFPs, along with the software used to produce them, are accessible via the Zenodo repository: https://zenodo.org/records/14832351. The data on exposure to UFPs, excess mortality calculations, and source sector attributions at the country, macro-region, and sub-region levels are provided in the supplementary data files.
