# Supplementary material for: Air quality and health implications of exposure to ultrafine particle pollution
Source: Cardiovasc Res. 2026 Jul 8;122(12):1660–71. doi: 10.1093/cvr/cvag136 (PMC13508697; doi:10.1093/cvr/cvag136)
Supplement: Supplementary file 1 [file cvag136_supplementary_data.zip › CVR-2025-2321_UFP_air quality_health_supplement_final.pdf]

Supplementary Materials for

Air quality and health implications of exposure to ultrafine particle pollution

Jos Lelieveld[1,2*], Pantelis Georgiades[2], Matthias Kohl[1], Yafang Cheng[3], Theodoros Christoudias[2], Jean Sciare[2], Mihalis Nicolaou[4], Constantine Dovrolis[4], Mohammed Fnais[5], Markku Kulmala[6], Andreas Daiber[7,8], Omar Hahad[7,8], Marin Kuntic[7,8], Thomas Münzel[7,8], Andrea Pozzer[1,2*]

[*]Max Planck Institute for Chemistry, Atmospheric Chemistry Department, Mainz, Germany. [2]The Cyprus Institute, Climate and Atmosphere Research Centre, Nicosia, Cyprus. [3]Max Planck Institute for Chemistry, Aerosol Chemistry Department, Mainz, Germany. [4]The Cyprus Institute, Computation-based Science and Technology Research Centre, Nicosia, Cyprus. [5]King Saud University, Department of Geology and Geophysics, Riyadh, Saudi Arabia. [6]Institute for Atmospheric and Earth System Research, University of Helsinki, Finland. [7]Department of Cardiology, University Medical Center of the Johannes Gutenberg University Mainz, Germany. [8]German Center for Cardiovascular Research, Partner Site Rhine-Mainz, Mainz, Germany.

* Corresponding authors. E-mail: jos.lelieveld@mpic.de (J.L.); E-mail: andrea.pozzer@mpic.de (A.P.)

Materials and Methods

Machine learning

A scarcity of global measurements limits the investigation of ultrafine particles (UFPs), particle number concentrations (PNCs) and their impacts on human health. Monitoring systems lack the spatial and temporal coverage to map UFP distributions at the appropriate resolution, i.e., to account for strong concentration gradients near sources and to characterise long-term exposure. The small size and dynamic nature of UFPs challenge traditional measurement techniques (Trechera et al., 2023). Estimating spatially distributed UFP concentrations has relied on land use regression and chemical transport models. The former can provide high spatial resolution but are only practical when extensive measurements are available, requiring local data and model tuning (Saha et al., 2021; Jones et al., 2020). Chemical transport models offer broader coverage but at a coarser spatial resolution, obscuring details near sources, especially in urban areas (Kohl et al., 2023).

To circumvent these limitations, we used machine learning (ML) to generalise particle number and UPF concentrations globally at high spatial resolution (Georgiades et al., 2025). Leveraging limited ground station measurements and incorporating Earth observational data (land use, urbanisation, built-up volume, satellite-observed pollution and emissions), we trained an Extreme Gradient Boosting (XGBoost) algorithm. This algorithm predicts annual average PNCs at 1 km global resolution over land and is integrated with a conformal prediction framework to quantify uncertainty (Johansson et al., 2014; Barber et al., 2021; Kim et al., 2020). It provides statistically robust 95% confidence (prediction) intervals (95% CIs) without prior assumptions of the data distribution. Additionally, we used SHAP (SHapley Additive exPlanations) to support the interpretation of results, i.e., enhancing understanding of predictions across different locations. This approach addresses data gaps and supports the comprehensive assessment of air quality and health implications.

ML algorithm

We used the XGBoost algorithm to calculate PNCs and subsequently estimate UFP concentrations. We selected this algorithm because of its computational efficiency, scalability, and recognized performance and flexibility. It employs ensemble tree-based learning, which is adept at handling various data types, resists outliers, and models complex, non-linear relationships without overfitting (Chen et al., 2016; Budholiya et al., 2022; Moore et al., 2022).

XGBoost implements gradient-boosted decision trees designed for speed and performance. Mathematically, the model is constructed stage-wise. The objective function in XGBoost includes a loss function to assess how well the model fits the data, along with a regularization term that penalizes the model’s complexity. XGBoost uses an additive training method, progressively incorporating new trees into the model. Each new tree is specifically trained to predict the residual errors left by the existing trees. This strategy enables the management of large datasets and high-dimensional feature spaces effectively, making it highly suitable for estimating UFP concentrations on a global scale.

Conformal prediction

To evaluate the model's predictive performance, we utilized the conformal prediction statistical framework to estimate the uncertainties of the results. Conformal prediction offers a method to

generate statistically valid prediction intervals that are independent of specific distribution assumptions, unlike methods such as Natural Gradient Boosting and Gaussian processes, which assume a normal data distribution. This assumption can be problematic with real-world datasets (Johansson et al., 2014). For our implementation, we employed the Model Agnostic Prediction Interval Estimator (MAPIE) library in Python 3.11, applying it to the XGBoost Regressor from the *xgboost* library.

Generally, conformal prediction involves training the base model and calculating prediction intervals using a holdout dataset. Given the limited data available in this study, we applied the jackknife+ after bootstrap method to enhance the robustness of our prediction intervals. This approach includes the following steps:

Bootstrap resampling: We resampled the training dataset multiple times (20 in this case) to create several bootstrap samples. The XGBoost regression model was trained separately on each sample.

Leave-one-out predictions: For each bootstrap sample, we performed leave-one-out (LOO) predictions, where each instance in the sample is excluded once during the predictions.

Nonconformity scores: We assessed the nonconformity of each prediction by comparing the LOO predictions with the actual values using mean-squared error. These scores measure how well the predictions conform to the observed data.

Interval calculation: We determined the bounds of the prediction intervals for new data points based on the distribution of nonconformity scores across all bootstrap samples and the desired confidence level ($\alpha = 0.05$ for a 95% confidence interval).

The jackknife+ after bootstrap method ensures that the coverage level (the proportion of observed data falling within the predicted confidence intervals) is higher than $1\text{-}2\alpha$ for a target coverage level of $1\text{-}\alpha$, without making prior assumptions about the data distribution, where α represents the confidence level (Kim et al., 2020; Barber et al., 2021).

To identify the optimal hyperparameters for the model, we initially set aside 10% of the dataset as a validation set, ensuring this part was not involved in parameter tuning. The remaining data was divided into 90% for training and 10% for testing. We employed GridSearchCV from scikit-learn to conduct a thorough search of the parameter space and evaluate model performance using the test set. The hyperparameters explored included the number of estimators (250), the number of parallel trees (1 to 15), maximum depth (10), learning rate (0.03), subsample ratio of training instances (0.75), and subsample ratio of columns for tree construction (0.75).

After identifying the optimal parameters, we assessed the model's effectiveness using the validation data. We trained the model on the full dataset to ensure it captured all relevant patterns and relationships. To evaluate the model's generalizability, we conducted a K-fold cross-validation analysis, partitioning the dataset into K folds (10 in this case). In each fold, 90% of the data was used for training and 10% for testing. Furthermore, spatial Leave-One-Out cross-validation (LOOCV) and temporal LOOCV were conducted to assess the model's performance in predicting unobserved locations and time periods. In each of the twenty cross-validation iterations (2000–2020), one year was omitted, and the model was trained using the remaining data. Fig. S1 illustrates the inputs (data sources), the spatial homogenization of the data, the methods—including uncertainty quantification—and the ML outputs generated in this work.

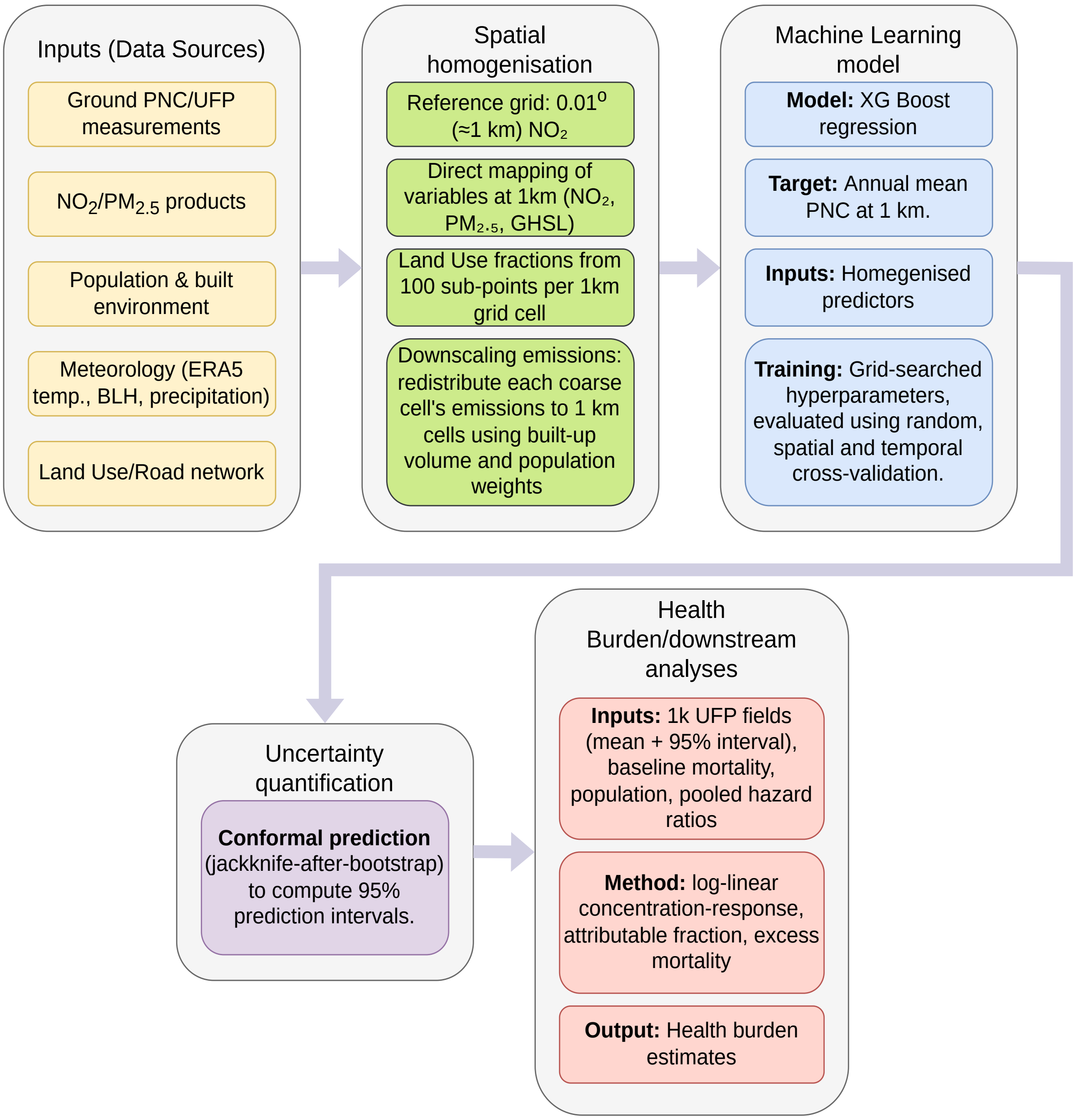

Figure S1. Schematic of the workflow related to our machine learning modelling of UFP exposure and the health burden estimates presented here.

Data sets

PNCs and UFPs. To acquire the target parameters for our ML model, we utilized a method that integrates data from various sources. First, we accessed the EBAS (https://ebas.nilu.no) and NOAA National Centers of Environmental Information (https://www.ncei.noaa.gov) databases to obtain particle size distribution and PNC data from ground stations across Europe and North America. For particle size distributions, we aggregated measurements of particulate matter with a diameter less than 100 nm to represent UFPs, while PNC data were derived directly, often used as a proxy for UFP concentrations. To ensure the data representativeness, we excluded stations with fewer than 150 unique days of data per year. We queried the databases for data for the period 2000 – 2020.

Further, we conducted an extensive literature review to complement the ground station data with information from published scientific articles that provided yearly averages for locations worldwide (Aalto et al., 2005; Kohl et al., 2023; Saha et al., 2021). The review enhanced the dataset by integrating findings from various geographical locations and monitoring networks. By synthesising data from the EBAS and NOAA databases and the scientific literature, we constructed a representative dataset for training and evaluating our ML model (Fig. S2; from Georgiades et al., 2025). The complete record comprised 836 yearly UFP datasets for different periods, collected from 155 locations.

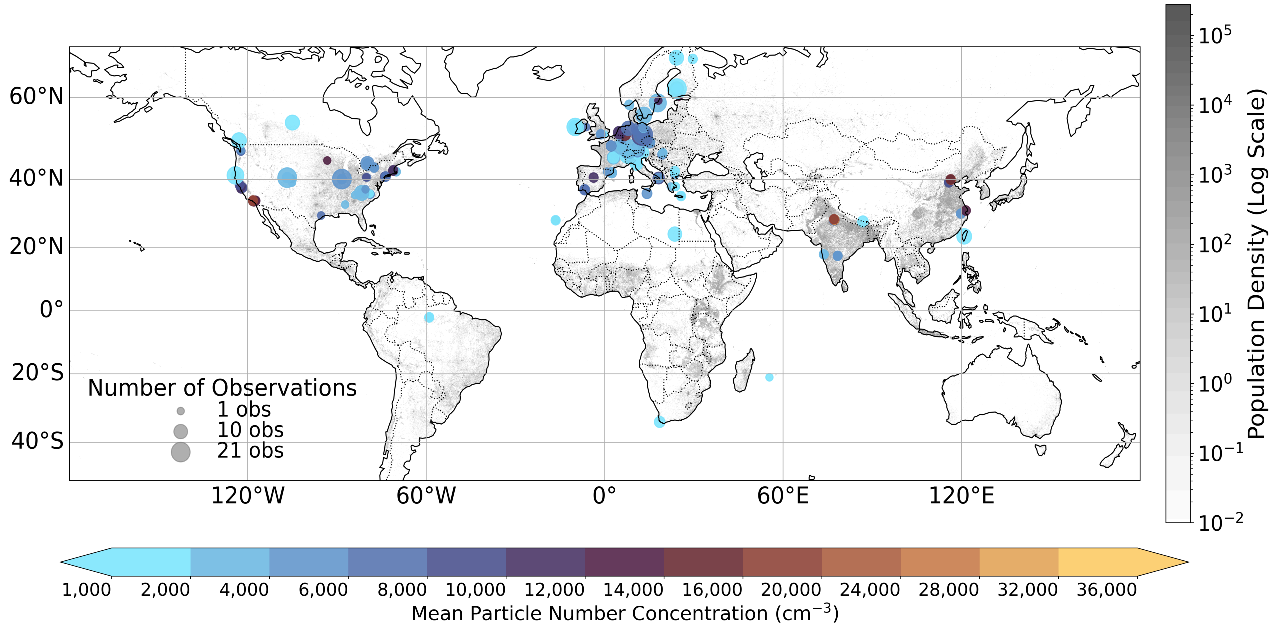

Figure S2. Locations of measurements included in the PNC dataset. Circle sizes indicate the number of observational datasets from each location (ranging from 1 to 21), while colours represent the mean particle number concentration (in cm^{-3}). The background grayscale indicates the global human population density per area of 1 km^2 on a logarithmic scale (from Georgiades et al., 2025).

Global human settlement layer. The Global Human Settlement Layer (GHSL) of the European Commission provides freely accessible data and tools for assessing human presence and activities. The global built-up volume (GHS-BUILT-V) dataset, utilised in 5-year increments and with a 1 km spatial resolution (Pesaresi and Politis, 2023), includes residential and non-residential buildings covering industrial and commercial complexes. Other datasets, such as human settlement (GHS-POP) (Schiavina et al., 2023a) and the degree of urbanisation (GHS-SMOD) (Schiavina et al., 2023b), were also used at the same temporal and spatial resolution. The GHSL datasets offer insights into anthropogenic activities and industrialisation indicators, such as areas where high built-up volume coincides with low population density, potentially indicating industrial areas or other high-emission activities. No temporal or spatial interpolations were performed; the nearest available year for each dataset was used, as these variables change minimally over time. Given the strong link between emissions and human activity, these datasets provide proxies for pollution sources.

Global NO_2 and $PM_{2.5}$. The global datasets of NO_2 and $PM_{2.5}$ were integrated into the feature set to assess the annual average concentrations of these pollutants (Anenberg et al., 2022; van Donkelaar et al., 2021). Each dataset has a spatial resolution of 0.01°, about equivalent to 1 km at the equator

and decreasing towards high latitudes, and a temporal resolution of one year. The datasets provide annual average concentrations of NO_2 and $PM_{2.5}$ for each grid cell. In part, created for epidemiological and health-burden research, these datasets align with the objectives of our study. The spatial grid was based on the NO_2 dataset's orthogonal latitude-longitude grid. The data cover a latitude range from 55°S to 68°N, following the geographical extent of the $PM_{2.5}$ dataset.

Pollution emissions. The gridded distribution data of global anthropogenic emissions from the Copernicus Atmosphere Monitoring Service (CAMS) were employed to derive combustion-related emissions information (Denier van der Gon et al., 2023). This dataset includes updated CAMS information up to 2023, accessed from the Copernicus Atmosphere Data Store (https://ads.atmosphere.copernicus.eu/cdsapp#!/home). To estimate UFP concentrations and assess anthropogenic impacts, proxies were derived from global emission inventories, incorporating yearly average emissions of black carbon (BC), carbon monoxide (CO), carbon dioxide (CO_2), and nitrogen oxides (NO_x).

The datasets provide sector-specific emissions and cumulative totals. The latter were selected for each emission type. Focus was given to emissions over land; thus, grid cells categorized as 100% "open sea" based on land cover data were excluded. We specifically considered emissions from combustion processes. Data were retrieved at a spatial resolution of 0.1° and monthly temporal resolution. Yearly averages per grid cell were calculated using the resample method in the Python *xarray* library. Spatial interpolation was then applied to redistribute emissions in each grid cell according to population density and built-up area.

Temperature. The data was derived from ERA5, the fifth-generation ECMWF reanalysis. This provides a proxy for meteorological conditions, with the 2m temperature (t2m) variable obtained from the Copernicus Climate Change Service (C3S) Climate Data Store (CDS). This data is available at a spatial resolution of 0.25° and an hourly temporal resolution (Hersbach et al., 2023). Temperature was chosen due to its role in atmospheric processes, including UFP dynamics through the photochemical oxidation of volatile organic compounds and nitrogen oxides (NO_x), as well as the phase state of semi-volatile reaction products (Kaur et al., 2022; Zhao et al., 2018). The dataset, containing Copernicus Climate Change Service information (2023) from the Copernicus Atmosphere Data Store (https://cds.climate.copernicus.eu), was processed using Python 3.11's *xarray* library to compute yearly averages for each grid cell, with no spatial interpolations applied during this procedure.

Boundary layer height. Like temperature the Boundary Layer Height (BLH) was also adopted from the ERA5 reanalysis dataset. BLH is related to the vertical mixing and dilution of pollution particles in the lower atmosphere, and thus, by incorporating BLH data, we account for the influence of atmospheric stability on particle concentrations near the surface. Shallow boundary layers, typically occurring during nighttime or winter conditions, lead to particle accumulation near the ground, while deeper boundary layers in summer and at low latitudes enhance vertical mixing and dilution (Li et al., 2017).

Precipitation. Precipitation is a relevant meteorological parameter for PNC prediction, as it governs particle removal through in- and below-cloud wet deposition processes. We have incorporated the total precipitation amount for each grid cell from the ERA5 reanalysis dataset to account for this removal pathway of atmospheric particles. Wet deposition is especially important in regions with

frequent rainfall events, where particle removal can substantially influence the annual average PNC (Hussein et al., 2018; Niu et al., 2023).

Road network. The Global Roads Open Access Data Set v1 (gROADSv1) is a comprehensive global road network database incorporating major roads and highways worldwide. We included this dataset as road traffic represents a primary source of ultrafine particle emissions in urban environments. The dataset provides detailed spatial information about road networks on a global scale by including roads and highways as lines in shapefile format. To convert to a gridded dataset, we calculated the number of roads intersecting every cell of the global grid as a proxy for capturing the traffic-related particle emissions. This information is helpful since vehicle exhaust emissions have been shown to create strong spatial gradients in particle number concentrations, with elevated levels typically observed near major roadways and traffic corridors (Wang et al., 2023).

Population. We included the global population dataset from WorldPop (www.worldpop.org) in our analysis (by the University of Southampton, UK). This dataset provides annual population counts with a spatial resolution of 1km globally. We acquired the data directly from the organization's website without altering temporal or spatial aspects. To ensure the required temporal coverage for our analysis, we retrieved data from 2000 to 2020. The WorldPop population counts dataset provides key information for our study, offering valuable insights into the spatial distribution of human populations across various regions.

Data homogenization

The NO_2 dataset, with a resolution of 0.01°, approximately 1 km at the equator, served as the baseline for creating a globally uniform gridded dataset. This data was used as a reference for standardizing the spatial resolution of other datasets, ensuring uniformity during training and inference. To incorporate land use data into this uniform dataset, 100 grid points within each 1 km grid cell were determined. For each land use class, the percentage coverage was extracted, resulting in seven distinct features.

Datasets with the same spatial resolution as NO_2, such as $PM_{2.5}$ and GHSL, were smoothly integrated into the uniform gridded dataset to maintain consistency without introducing discrepancies. A redistribution procedure was applied to address the resolution difference between the emissions dataset (10 km grid) and other datasets (1 km grid). This procedure preserved the total emissions within each 10 km grid cell while redistributing them to a higher resolution. Redistribution was carried out by linearly weighting emissions based on population and built-up volume, ensuring alignment with other datasets, as described by Kohl et al. (2023), and summarized in the section “Downscaling UFP Concentrations” below.

Evaluation of ML model results

We assessed the performance of the XGBoost model using several validation strategies to ensure robust predictions of global PNC distributions. An extensive grid search was conducted to determine the optimal hyperparameter configuration. The final model employed 250 estimators with a single parallel tree, a maximum tree depth of 10, and a learning rate (η) of 0.03. Both the subsample ratio of training instances and the subsample ratio of columns were set to 0.75. To further evaluate model stability, we conducted a sensitivity analysis based on the fitted models generated during the exhaustive hyperparameter search, using the held-out data portion for validation in each iteration. Using these optimized parameters, Fig. S3 illustrates the predictive performance of the model. The

conventional train–test split evaluation achieved a correlation coefficient (r^2) of 0.90 and a Mean Absolute Error (MAE) of 1,336 cm^{-3}, whereas the 10-fold cross-validation yielded slightly improved results, with an r^2 of 0.91 and an MAE of 1,025 cm^{-3}.

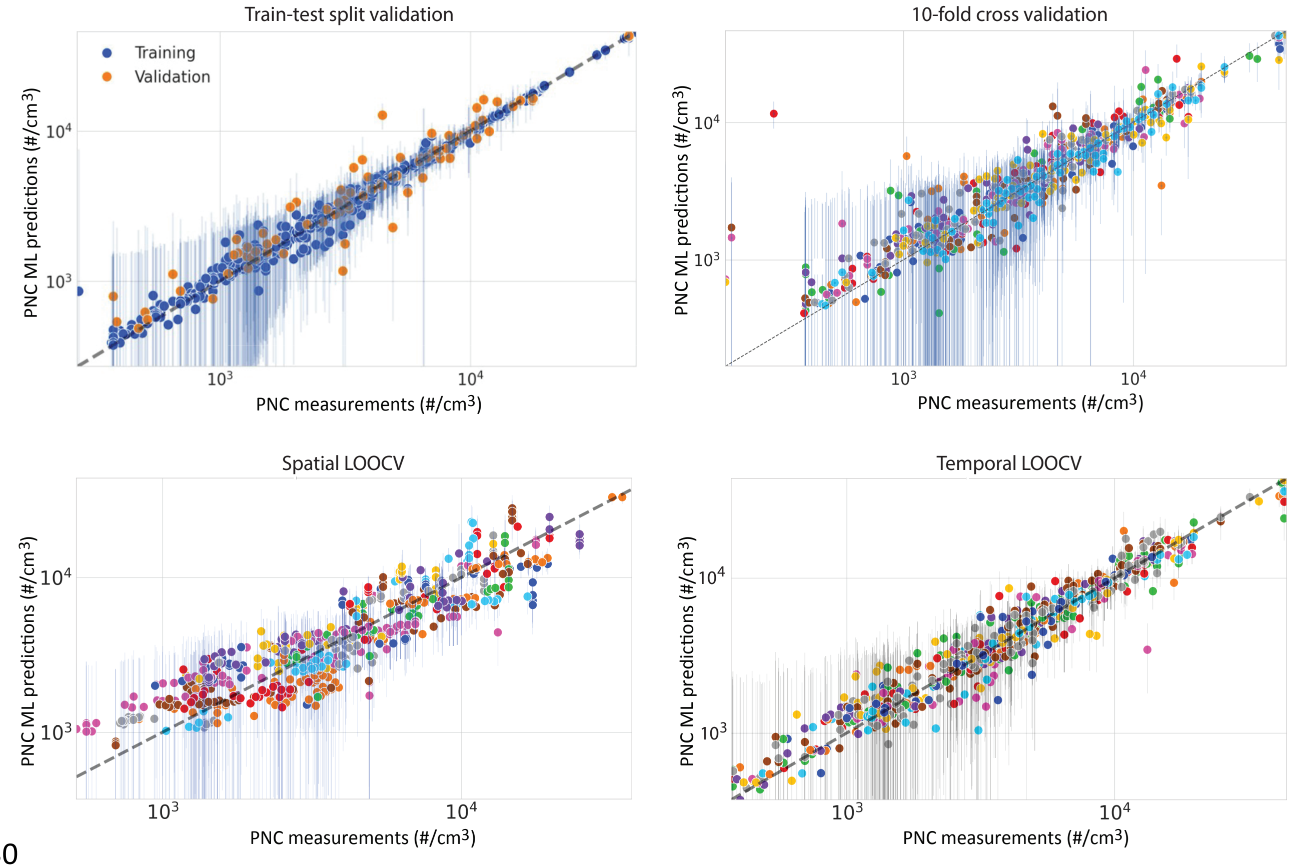

Figure S3. Comparison of PNC observational datasets with ML model predictions. Upper left: Blue bullets represent the training dataset (90%), and orange ones the evaluation set (10%), not seen during training. Upper right: Predicted versus observed PNC values from 10-fold cross-validation, showing only out-of-fold (held-out) predictions for each fold. Lower left: Predicted PNCs from spatial Leave-One-Out Cross-Validation (LOOCV). Lower right: Predicted PNCs from temporal LOOCV where full years have been held out. Vertical lines on each point represent the 95% confidence intervals (from Georgiades et al., 2025).

To evaluate the model's capability to predict PNCs at unseen locations and time periods, we applied both spatial and temporal Leave-One-Out Cross-Validation (LOOCV) (Fig. S2). In the spatial LOOCV approach—where entire measurement stations are withheld—the model achieved an r^2 of 0.77 and an MAE of 2,839 cm^{-3}. This lower performance highlights the inherent difficulty of spatial extrapolation to entirely new sites, particularly given the limited global coverage of measurement stations.

The reduced accuracy observed in the spatial LOOCV underscores a key limitation in global PNC estimation: the uneven spatial distribution of ground-based monitoring networks. As shown in Figure S2, most monitoring stations are concentrated in Europe and North America, leaving substantial data gaps across low- and middle-income regions such as Africa and South America.

These data-sparse areas exacerbate extrapolation errors, a major challenge in global air pollution modelling. Insufficient monitoring coverage in developing regions introduces considerable uncertainty in exposure assessments and compromises model generalizability, leading to lower predictive accuracy where ground validation is scarce.

In contrast, the temporal LOOCV, in which entire years were withheld, exhibits strong predictive performance with an r^2 of 0.87 and an MAE of 1,740 cm^{-3}. This comparatively higher temporal accuracy indicates that the model more effectively captures interannual variations than spatial patterns, likely due to the more consistent nature of the temporal processes influencing PNC distributions. The strong temporal generalization further suggests that the selected predictor variables successfully represent the dynamic mechanisms controlling particle concentrations, even when applied to previously unseen years.

Percentage errors remain relatively stable across validation approaches, ranging from 23% for the traditional test set to 32% for the spatial LOOCV. The spatial LOOCV yields a lower prediction bound of approximately 1,000–1,500 cm^{-3}, primarily reflecting the scarcity of training stations at higher latitudes, which were excluded as they are located outside the defined latitudinal range of input variables. These findings represent the current state-of-the-art in global PNC prediction, given the novelty of long-term PNC observations and the limited availability of measurement data worldwide. Despite these constraints, the model demonstrates robust extrapolation capacity to new locations, offering a valuable framework for global-scale PNC estimation.

Model performance also varies notably across population density categories. In densely urbanized regions (>1,900 people km^{-2}), where both annual and global mean PNCs are highest (14,992 cm^{-3}), the absolute uncertainty is greatest, with a mean 95% CI of 3,715±182 cm^{-3}. However, due to the high concentration levels in these areas, this corresponds to a relatively smaller percentage error of 29±2%. Suburban regions (250–800 people km^{-2}) show intermediate PNC values, with a global mean of 6,360 cm^{-3} and a percentage error of 35±3%. While rural areas (<250 people/km^2) are associated with the smallest absolute 95% CI (1,852±56 cm^{-3}), they have the highest percentage error of 91±3% associated with their low mean PNCs (2,606 cm^{-3}).

Although percentage errors are higher in rural areas, this is not critical from an exposure assessment perspective, as these regions typically combine low population densities, relatively low PNCs, and minimal associated health impacts. Nevertheless, episodic pollution events—such as large wildfires—can cause acute spikes in PNC. These episodes are often underrepresented in long-term averages. This underrepresentation highlights the need for enhanced measurement coverage in under-monitored regions. While satellite-based observations have advanced efforts to fill global monitoring gaps, they too face challenges—particularly related to regional validation and context-specific uncertainties. Consequently, predictions for data-poor regions should be interpreted with caution, as the model may not fully capture local emission dynamics, meteorological conditions, and atmospheric processes unique to those areas. To better assess regional uncertainty, the data users are encouraged to consult the model's conformal prediction coverage intervals, which adaptively expand in regions with limited training data density.

The variability in model performance gains additional significance in the context of global demographic shifts. According to WorldPop population data, the proportion of people living in suburban and urban areas increased by more than 5% in the past two decades, coinciding with the global population growth from 6 to 8 billion. This trend underscores the growing importance of

accurately modelling air pollution exposure in urban and suburban environments—where both population density and PNC/UFP concentrations are highest. Encouragingly, the relatively low percentage errors observed in these areas support reliable exposure assessments for an expanding share of the global population. While rural regions exhibit higher percentage errors, their low population densities and lower pollution levels mitigate the overall impact of these uncertainties on population-scale exposure estimates. Nevertheless, expanding measurement efforts in rural settings remains essential to improve model generalizability across diverse environmental conditions.

Conformal prediction is key in providing robust uncertainty quantification despite the limited spatial coverage of the global monitoring network. Unlike traditional methods that rely on strict distributional assumptions, conformal prediction produces distribution-free prediction intervals by leveraging the exchangeability of training and test data. Although this assumption can be theoretically violated in real-world contexts—such as through gradual temporal trends in emissions or evolving monitoring networks—our analysis mitigates these risks in two ways. First, the use of yearly-averaged data reduces sensitivity to short-term fluctuations, minimising the impact of gradual temporal trends on exchangeability. Second, empirical validation demonstrated that the conformal intervals maintained approximately 95% coverage across held-out test sets spanning multiple regions and years, with no notable degradation in performance.

Importantly, the prediction intervals adaptively widen in data-sparse regions, accurately reflecting increased uncertainty and demonstrating robustness to minor violations of exchangeability. While abrupt temporal shifts (e.g., sudden emission reductions following policy interventions) could pose greater challenges, such effects were not observed in our analysis. The practical robustness of this framework is further supported by strong temporal cross-validation performance (r^2 = 0.87), where conformal prediction achieved near-target coverage.

UFP and PNC measurement data

To estimate UFP concentrations from total PNC measurements, we analyzed particle size distributions from the EBAS database (Georgiades et al., 2025). We find that UFPs consistently dominate the total particle number concentrations, with a mean contribution of 91%, in support of previous work (Presto et al., 2021). To characterise the distribution of UFP fractions and quantify uncertainties, we fitted a Beta probability distribution to the normalised data (scaled to a range of 0 to 1), which is suitable for modelling fractions and bounded data. It provides a complete distribution of the underlying probability, which can be skewed or asymmetric. The shape parameters α and β allow the distribution to take various forms; here, it is right-skewed and unimodal, with a peak near 1. The fitted Beta distribution has shape parameters α = 18.75 and β = 1.89. From this fit, we derived the UFP fraction, with a mean value of 0.9082 across all data, and a variance of 0.0054 (see Figure S4).

The small variance indicates that the data are compactly clustered, i.e., close to each other and to the mean, indicating consistency and robustness of the relationship between UFPs and PNCs. Furthermore, it confirms that PNC measurements are a reasonable proxy for UFPs, with only a slight overestimation of their concentrations (~10%). Using the standard error of the mean, we derive a bounded confidence interval (Beta-distribution–based percentile interval) of 0.9082 (CI95% 0.7866 – 1.0299). Using this function, we derived UFP concentrations from the PNC data and included the uncertainty ranges in the evaluation of mortality burden estimates.

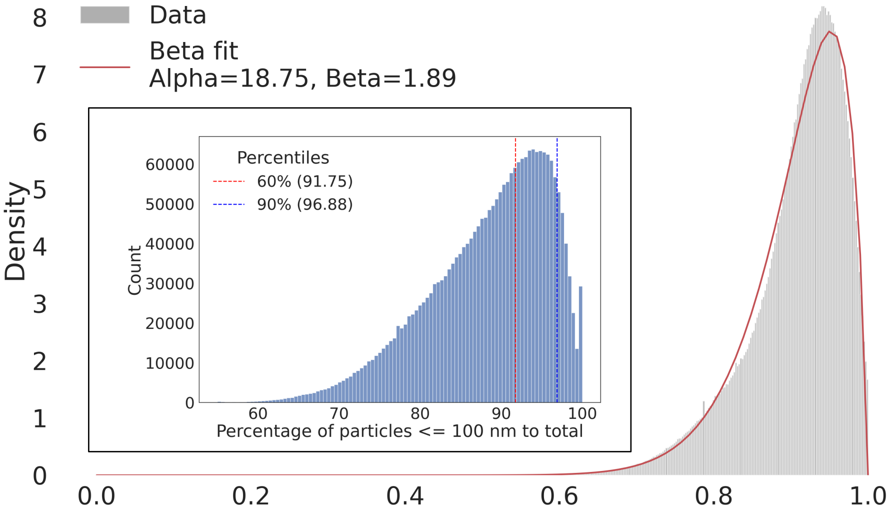

Figure S4. Beta distribution of the normalised UFP fractions (0 – 1) relative to the total PNCs. The outer panel shows the Beta distribution, capturing the variability in UFP fractions. Inset: histogram of the percentage of particles with diameters less than 100 nm relative to the total PNC, with vertical dashed lines indicating the 60% (red) and 90% (blue) percentiles (from Georgiades et al., 2025).

Explainability

To analyse the operations of the ML model, we employed the SHAP (SHapley Additive exPlanations) method. Shapley values, derived from cooperative game theory, evaluate the individual contribution of each input feature to a specific prediction, allowing us to identify and quantify the features that most significantly influence the model output (Lundberg et al., 2017). The essence of SHAP lies in comparing the model prediction for a given data point to predictions made under various hypothetical scenarios in which specific features are "masked out." By aggregating these contributions, SHAP assigns an attribution value to each feature that reflects its impact on the final prediction (Lundberg et al., 2020).

The model has been retrained on all feature subsets. Each feature's importance value is determined by its effect on the model output when included. To compute this effect, two model versions were trained: one with the feature included and one without. The prediction difference between these two models is computed for each input feature. Since the effect of removing a feature depends on other features in the model, these differences are calculated for all permutations of the feature. SHAP values, which represent feature attributions, are then computed as the weighted average of all possible differences (Lundberg et al., 2017; Pezoa et al., 2023).

A positive SHAP value indicates the feature enhances the model prediction, while a negative value indicates the opposite. The magnitude of the value reflects the strength of this influence (Nohara et al., 2021). We used the SHAP library in Python and the TreeExplainer method to create beeswarm and waterfall visualisations to help clarify feature attributions in the model and their impact on individual predictions (Lundberg et al., 2020). SHAP is a model-agnostic framework that calculates feature contributions to specific predictions. The TreeExplainer method uses tree-based ML to determine these attributions. It constructs decision trees to simulate the original model operation, and by analysing how each feature affects the data splits in these trees, it assesses each feature's contribution to the final prediction. Fig. S5 shows the features that most significantly influence the model output.

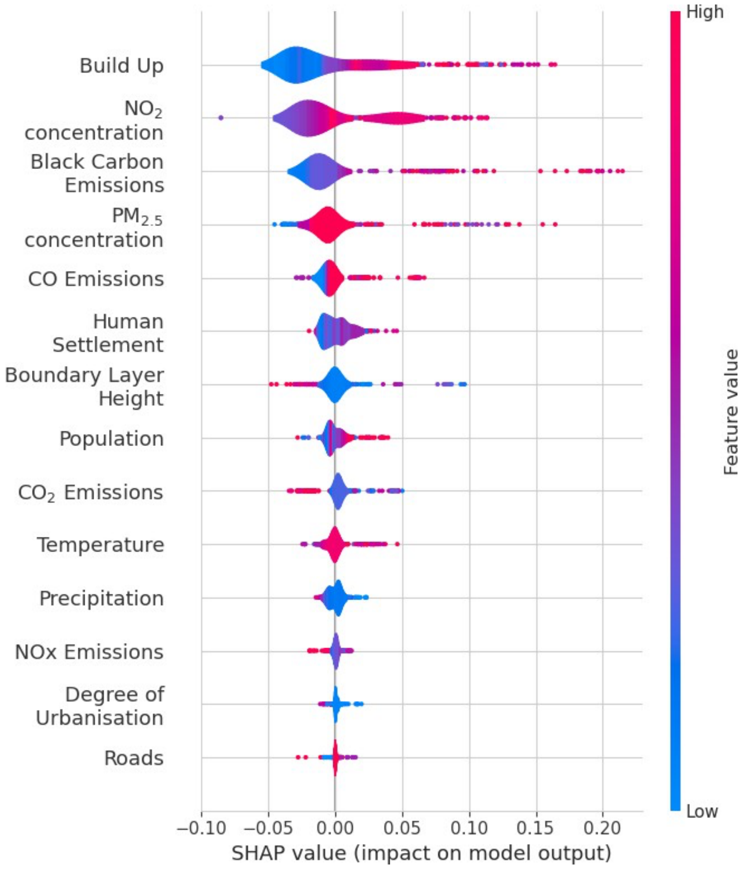

Figure S5. Feature importance based on SHAP values. Beeswarm plot, illustrating the influence of each feature on model predictions, where each point represents a single prediction. Features are ordered by their absolute SHAP values, with higher values indicating stronger influence on PNC predictions. The colours represent the feature values (blue for low, red for high) (Georgiades et al., 2025).

Such beeswarm plots display the distribution of SHAP feature attributions across all instances in the dataset. Features with a broad spread of SHAP values across the y-axis have a more significant impact on the model, while those clustering around zero have a minimal effect. This visualisation offers insights into how individual features interact and collectively influence the model predictions for specific instances. For details, we refer to Georgiades et al. (2025), presenting all datasets. Interestingly, road networks have only a minor impact. The reason is that road traffic is captured by other model features, particularly NO_2 concentrations and BC emissions, both of which are commonly used as proxies for traffic-related air pollution. Further, the static nature of this feature may not capture the dynamic nature of traffic emissions, which vary in space and with time.

Sources of uncertainty

We employed a novel data-driven framework to predict PNCs and UFPs globally at high spatial resolution. While the model demonstrates strong performance, several sources of uncertainty must be considered. These can be broadly categorised into data-related, model-related, and prediction-related uncertainties.

Data-related uncertainty

The main source of data uncertainty stems from the limited spatial coverage of ground-based measurements, particularly in regions with significant emission sources. The sparse distribution of PNC and UPF monitoring stations—especially across low- and middle-income countries undergoing rapid industrialisation and urbanisation—introduces sampling bias. This is most evident in Africa, South America, and parts of Asia, where data availability remains low despite substantial contributions to global emissions.

Most training data originate from Europe and North America, with limited representation from Asia and almost none from the Southern Hemisphere. Although the dataset captures diverse environmental conditions across multiple climates and land-use types, model performance in underrepresented regions should be interpreted cautiously, as local emission profiles and meteorological patterns may differ substantially. Spatial cross-validation helps mitigate but cannot entirely eliminate these extrapolation risks. Expanding ground monitoring networks and promoting open-access data sharing remain essential for improving model generalizability and reducing global exposure uncertainty.

Model-related uncertainty

The XGBoost model's ensemble and regularisation mechanisms enable robust predictions and effective handling of moderately sized datasets. Cross-validation results indicate that the model maintains stability even in data-scarce regions, with adaptive prediction intervals that widen appropriately according to uncertainty. The spatial LOOCV value of r^2 of 0.77, though lower than the random cross-validation value (r^2 = 0.91), remains consistent with prior global exposure models. For instance, land-use regression and hybrid models commonly achieve lower spatial LOOCV values, yet have successfully supported epidemiological cohort analyses.

Nonetheless, extrapolation to poorly monitored regions remains a challenge for all global-scale models. Integrating complementary approaches—such as hybrid modelling that combines machine learning with process-based chemical transport models, or the inclusion of satellite and low-cost sensor data—may enhance spatial representativeness. Importantly, our integration of conformal prediction enables explicit identification of regions with expanded uncertainty intervals, signalling where exposure estimates warrant cautious interpretation.

Prediction uncertainties

Prediction uncertainty arises from both aleatory (natural variability) and epistemic (knowledge-based) sources. These uncertainties are most pronounced in areas with environmental conditions that are poorly represented in the training data or during temporal periods absent from the dataset. The conformal prediction framework used here provides distribution-free uncertainty quantification, ensuring valid coverage even when extrapolating beyond observed conditions.

Spatial LOOCV results (r^2 = 0.77) indicate greater uncertainty in under-monitored regions than in random cross-validation (r^2 = 0.91). Urban areas exhibit the lowest relative uncertainties, benefiting from comparatively dense monitoring coverage, whereas rural regions show higher percentage errors—though these are less critical for exposure assessment due to their lower population densities and PNC levels.

To further reduce predictive uncertainty, it is crucial to expand both the spatial and temporal coverage of PNC and UFP measurements globally and to make these data openly accessible. Doing so will enhance model performance across diverse environments, strengthen exposure assessments, and support more equitable representation of air pollution impacts worldwide.

Meta-analysis of hazard ratios

The left panel of Fig. S6 shows the hazard ratios (*HRs*) from four large cohort studies: the largest one by Pond et al. (2022) with 617,966 participants, the smaller one by Pond et al. (2022) with 396,470 participants, the very large one by Bouma et al. (2023) with 10,735,734 participants, and the large

one by Lloyd et al. (2024) with 1,544 million participants (in total about 13.3 million participants). The left panel presents the pooled hazard ratio and its 95% confidence interval (95% CI), obtained from a random-effects meta-analysis using the DerSimonian–Laird estimator. The analysis indicates a 5.7% increase in all-cause mortality per 10,000 particles/cm³ increment in the ambient UFP concentration. The heterogeneity statistics yield τ^2 = 0.000218, Cochran Q = 13.587, p(Q) = 0.0035, and I^2 = 77.9%. The latter high percentage is related to the relatively small number of cohorts, and means that nearly 78% of the total variability between study estimates is due to heterogeneity rather than sampling error, indicating substantial between-study variability.

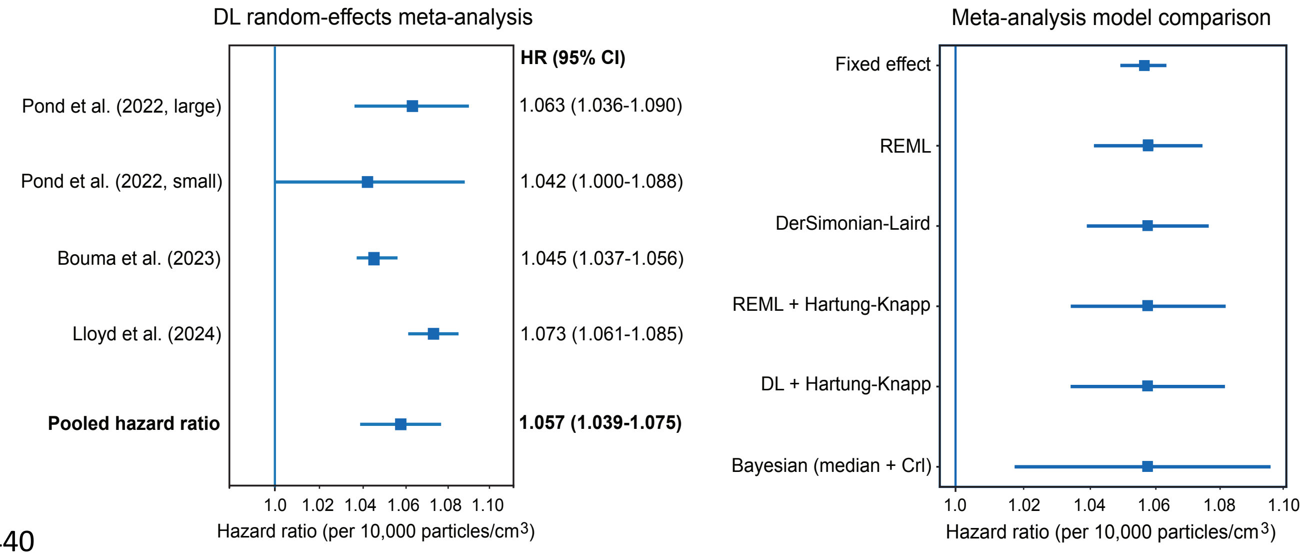

Figure S6. Forest plots, showing the hazard ratios for all-cause mortality from different cohort studies and the pooled *HR* (left), and the results from a sensitivity study applying a fixed-effect and several random-effect pooling methods (right).

For this reason, we performed a sensitivity analysis, using alternative *HR* pooling methods that are more/less affected by the number of studies (Restricted Maximum Likelihood, REML, DerSimonian–Laird, Hartung–Knapp, and Bayesian random-effects), which show almost identical pooled estimates (*HR* is 1.056 – 1.058 per 10,000 particles/cm³). This indicates that results are robust to the choice of meta-analytic model (Fig. S6, right panel). The various methods differ only in their 95% confidence or credible intervals.

Fig. S7 presents a graphical representation of the relationship between UFP concentrations and the attributable fraction (*AF*) of all-cause mortality across the four cohort studies adopted here. As UFP levels increase from 0 to 50,000 particles/cm³, the *AF* increases approximately linearly. Individual curves represent estimates from Pond et al. (2022, small and large cohorts), Bouma et al. (2023), and Lloyd et al. (2024), with the latter showing the steepest increase. Black solid and dashed lines depict weighted average (pooled *HR*) estimates across studies and the 95% CIs, respectively. Note that we do not adjust *HRs* at very high UFP concentrations, which is sometimes applied for $PM_{2.5}$, for example, in integrated exposure response functions (Pozzer et al., 2023). Not applying such an adjustment could potentially lead to an overestimation of excess mortality at the upper UFP concentration range.

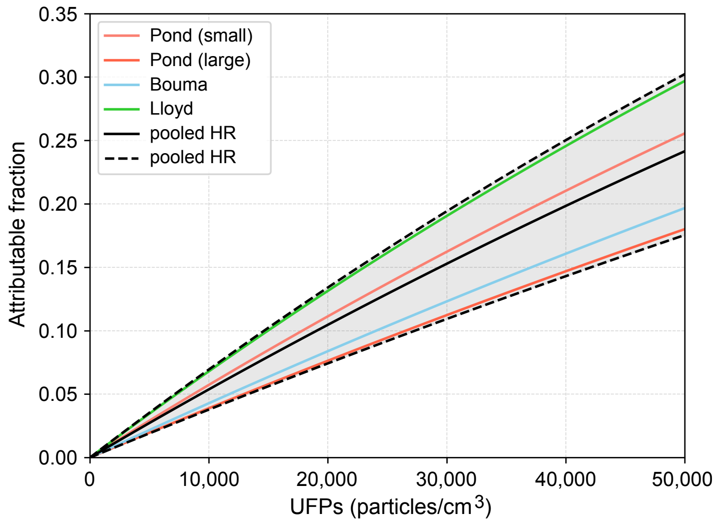

Figure S7. Line graph showing the attributable fraction (*AF*) of all-cause mortality as a function of exposure to UFP concentrations, with 95% CIs (shaded area).

EMAC model and emissions

We have developed an Earth system model simulating atmospheric chemistry and climate (EMAC) (Jöckel et al., 2016; Pozzer et al., 2022). The anthropogenic emissions of trace gases and particles used as model input have been adopted from the Community Emission Data System (CEDS) (McDuffie et al., 2020). Source sectors include fossil energy production, industry, land transport, shipping, aviation, domestic energy use, waste incineration, agriculture, solvent production and use. The CEDS emission data have been produced at a geographical resolution of 0.5°. In addition, we employed the Emissions Database for Global Atmospheric Research (EDGAR) at 0.1° resolution latitude and longitude for 2015 for downscaling (Crippa et al., 2020). Pollution emissions from forest and savanna fires and agricultural waste burning are based on satellite remote sensing data products and emission factors from Andreae (2019). Natural emissions, e.g., sea spray, desert dust and biogenic volatile organic compounds (VOC) from the vegetation are computed interactively depending on meteorological and soil conditions (Guenther et al., 2012; Klingmüller et al., 2018). The emission size spectrum of primary aerosol particles depends on the source sectors, according to Paasonen et al. (2016) and optimized according to measurements of size distributions (Kohl et al., 2023). We have used the UFP diameters to translate the emissions in the inventories, expressed in mass, to the UFP number concentrations.

Atmospheric chemistry and aerosols

EMAC simulates gas-phase and heterogeneous chemistry through multiple submodels that account for the photochemical oxidation of natural and anthropogenic emissions, including a comprehensive account of VOC such as alkanes, alkenes, terpenes (isoprene and lumped monoterpenes) (Sander et al., 2019). The gas- and particle-phase chemical mechanisms have been coupled through a submodel that simulates the organic aerosol composition and chemical evolution based on volatility (Tsimpidi et al., 2014). It accounts for primary and secondary emissions from fossil and biofuel combustion and biomass burning, as well as photooxidation during transport, which influences the phase state of the aerosol particles.

The coupling involves partitioning between the gas and particulate phases following a thermodynamic equilibrium approach (Pozzer et al., 2022). A similar methodology is applied to inorganic compounds and water uptake (Fountoukis and Nenes, 2007). Aerosol microphysical processes are computed with a sub-model that describes seven interacting lognormal size modes (four hygroscopic and three non-hygroscopic) (Pringle et al., 2010). The particles are internally mixed within each size mode, but they can transfer between modes (externally mixed) through condensational growth, evaporation and coagulation. The size spectrum encompasses the nucleation (<5 nm radius), Aitken (5–60nm), accumulation (60–700 nm) and coarse (>700 nm) modes. The smallest mode particles are formed by binary and ternary nucleation, pure organic nucleation, and nucleation from VOC oxidation products and sulfuric acid (Riccobono et al., 2014; Kirkby et al., 2016, 2023; Ehrhart et al., 2018).

The EMAC model also accounts for ion-induced nucleation via ion-pair production from galactic cosmic rays and the decay of radon emitted from soils (Kirkby et al., 2016, 2023). Physical removal processes involve dry deposition, gravitational sedimentation, and wet deposition through nucleation and impaction scavenging by cloud and rain droplets (Kerkweg et al., 2006; Tost et al., 2006). Evaluation of the aerosol simulations with the EMAC model against measurements has been documented previously, including black and organic carbon aerosols that dominate the sub-micrometre and UFP fractions (Chowdhury et al., 2022). A comprehensive, recently updated evaluation of atmospheric crustal compounds, black and organic carbon, aerosol optical depth and inorganic compounds is presented by Pozzer et al. (2022).

Downscaling UFP concentrations

Since the relatively coarse grid resolution of the EMAC model does not do justice to concentration gradients near strong sources of UFPs, particularly of primary particles near areas with heavy traffic and industrial emission hotspots, we downscaled the simulation results in two steps, first to 0.5° and then to 0.1° latitude and longitude (~10 km × 8 km, respectively, at mid-latitudes). Observation-guided downscaling was achieved by redistributing the model-grid-box-average UFP concentration according to the anthropogenic source sectors, available at higher resolution in the CEDS and EDGAR emission inventories (Kohl et al., 2023). UFPs in the model are defined as particles in the size fraction 3 nm to 100 nm, the former based on the lower size limit of most measurements.

Based on the ratios of modelled to observed UFP concentrations, relative anthropogenic emissions (x) were defined for model grid cells with strong particle-pollution sources (>2.0 10^6 m^{-2} s^{-1}) to identify areas with potentially steep gradients in UFP concentrations. A linear relationship between the observed and modelled particle number concentrations (N_o and N_m, respectively) was applied to redistribute the particles toward the source areas, successively in the 0.5° and 0.1° inventories. The function $N_o/N_m = c\,(x–1) +1$ was used, where x represents the relative anthropogenic emissions, i.e., the local anthropogenic particle number emissions relative to the average emissions of the grid box at coarser resolution; and $c = 0.13$ and $c = 0.15$ are the coefficients for CEDS and EDGAR, respectively (derived from least square fitting against observations). This procedure conserves the number and mass concentrations within the model grid boxes and redistributes particles only toward source regions. Results are presented in Fig. S.8 for the Los Angeles area in California and the Belgrade (Serbia) area in eastern Europe, respectively.

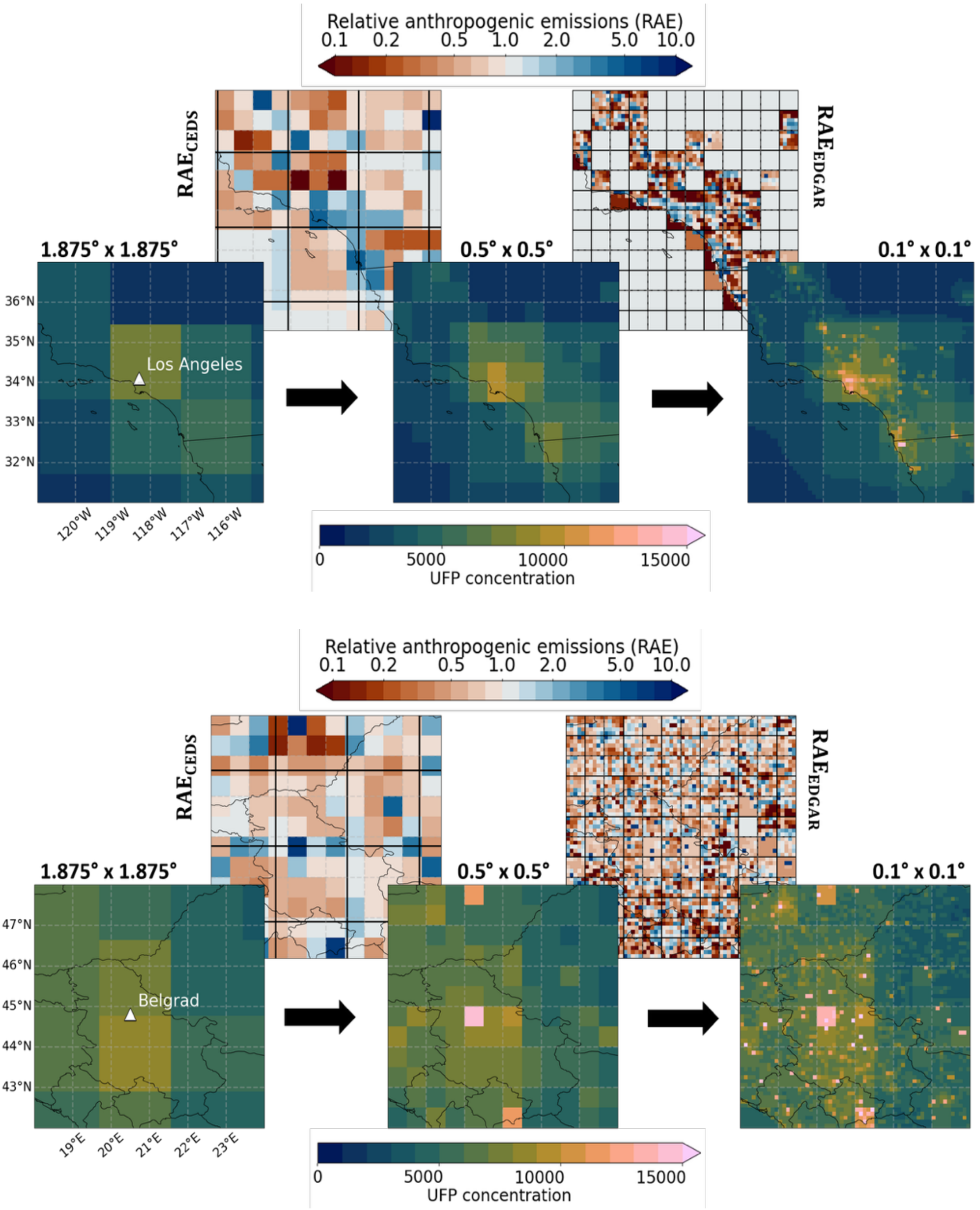

Figure S8. Illustration of UFP downscaling. The procedure employs anthropogenic particle number emissions from the CEDS (0.5°) and EDGAR (0.1°) inventories. The figures present examples of Los Angeles and Belgrade, in the western US and eastern Europe, and their surroundings. Relative anthropogenic emissions (RAE) represent the fractions of anthropogenic particle emissions from the CEDS and EDGAR inventories relative to the average over the model grid cells (top two panels). The two-step redistribution of modelled UFP concentrations within grid cells, maintaining the total mass and number concentrations, is shown in the bottom three panels.

Figure S9 compares the downscaled UFP model results with annual measurements from 60 measurement stations in various geographic regions (Europe, North America, India, China, and remote, mostly high-latitude locations) for which measurement data were available during the same period.

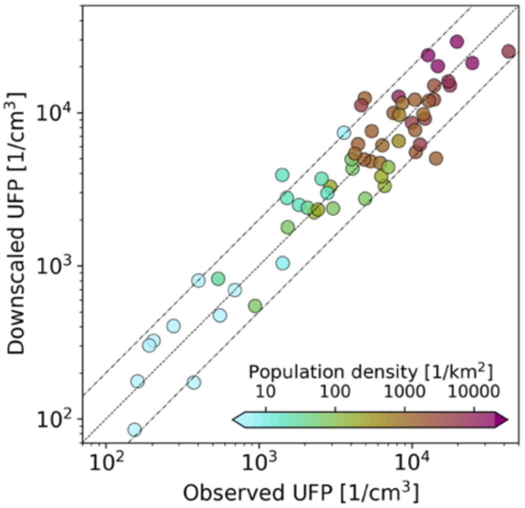

Figure S9. Observed and downscaled UFPs. Symbols refer to measurement stations in different geographic regions. The logarithmic correlation coefficient r^2 = 0.95 (the linear r^2 = 0.99), the slope of the linear fit is 1.022, and the RMSLE = 0.43. The colour scale indicates the population density at the measurement stations (0.1° resolution).

Chemical composition of UFPs

Figure S10 presents the EMAC model-calculated chemical composition of UFPs at the Earth's surface. Black carbon (BC) and a fraction of organic aerosols are primary, and ammonium sulfate, nitrate and part of the organics are secondary species. It illustrates the important role of BC particles, which are directly emitted as UFPs (primary), and secondary organic aerosol (SOA) compounds in populated and anthropogenically polluted regions. Primary organic aerosols (POA) and SOA compounds in UFPs are important in biomass-burning regions, including Canada and Siberia in the extra-tropics, as well as tropical South America and Central Africa.

The SOA fraction of UFPs is also significant over forested regions, both in the tropics and extra-tropics. Over the oceans, the UFP composition is dominated by secondary inorganic compounds, notably ammonium bisulfate and SOA (from natural emissions), and nitrate (influenced by shipping emissions) related to new particle formation, i.e., particle nucleation and growth. The model results in Fig. S8 show that UFPs are dominated by carbonaceous particles, unlike $PM_{2.5}$, which additionally contains significant fractions of inorganic and mineral compounds. The spatial distributions can differ considerably and partially correlate inversely, especially in non-polluted environments where UFPs depend more strongly on new particle formation.

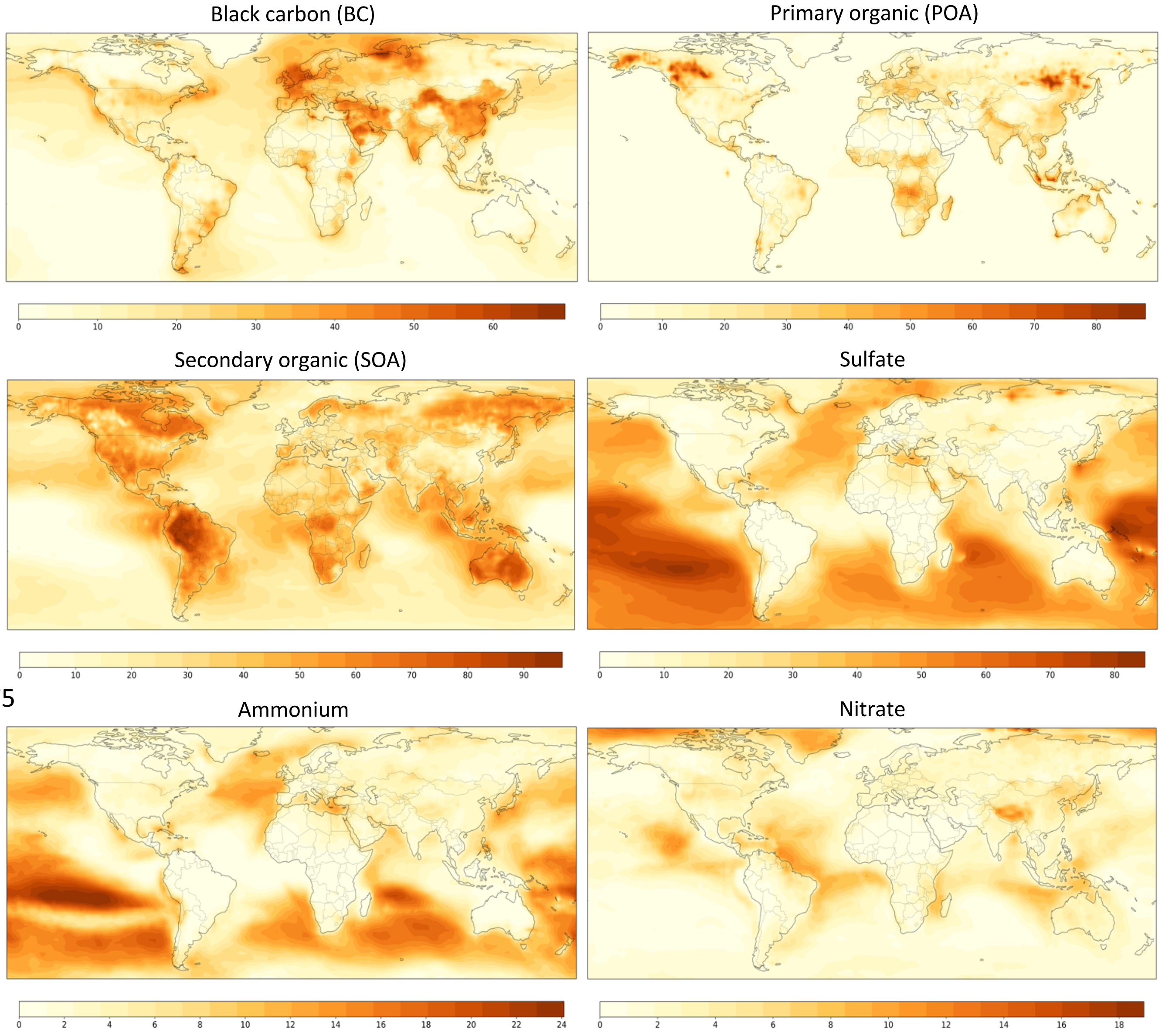

Figure S10. Model calculated annual mean mass fractions of UFP components (in percent – note scale differences). The main anthropogenic source sectors are energy production from fossil fuels, industry, land traffic and domestic energy use.

Data availability

The global results for PNCs and UFPs, as well as the software used to generate them, are available at 1 km grid resolution via the Zenodo general-purpose research output repository (https://zenodo.org/records/14832351). The results of exposure to UFPs, the excess mortality calculations, and the attribution to source sectors at the country, macro-region, and sub-region levels are available in the supplementary data files. These regions follow the classification of the Global Burden of Disease studies (Fig. S11).

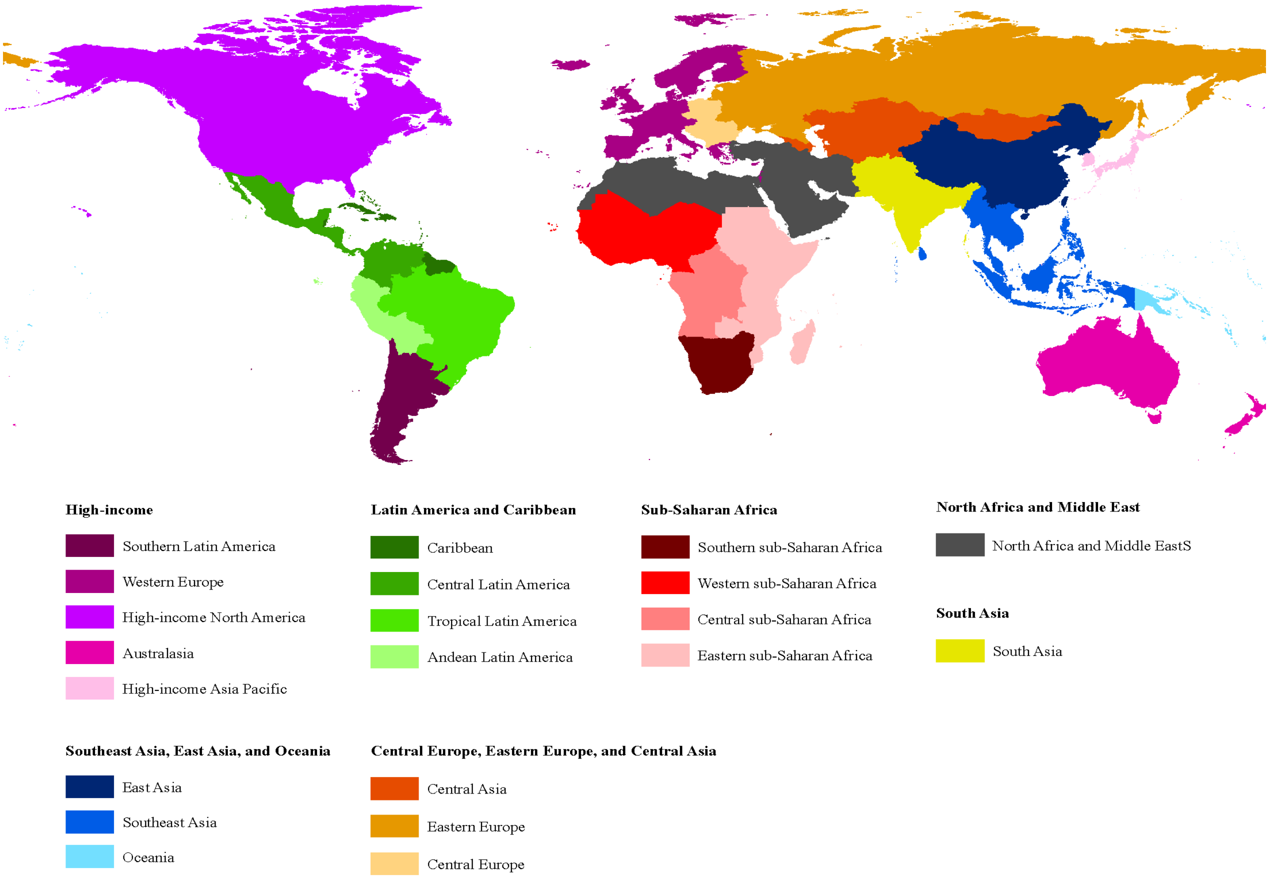

Figure S11. Macro-regions (7) and sub-regions (21), as defined by the Global Burden of Disease studies (GBD, 2020).

References

Aalto P et al. Aerosol particle number concentration measurements in five European cities using TSI-3022 condensation particle counter over a three-year period during health effects of air pollution on susceptible subpopulations. *J Air Waste Man Assoc* 2005;**55**:1064–1076.

Andreae M O. Emission of trace gases and aerosols from biomass burning – an updated assessment. *Atmos Chem Phys* 2019;**19**:8523–8546.

Anenberg SC et al. Long-term trends in urban NO_2 concentrations and associated paediatric asthma incidence: estimates from global datasets. *Lancet Planet.Health* 2022;**6**:e49–e58.

Barber RF, Candès EJ, Ramdas A, Tibshirani RJ. Predictive inference with the jackknife+. *Ann Stat* 2021;**49**: 486–507.

Bouma F, et al. Long-term exposure to ultrafine particles and natural and cause-specific mortality. *Environ Int* 2023;**175**:107960.

Budholiya K, Shrivastava SK, Sharma V. An optimized XGBoost based diagnostic system for effective prediction of heart disease. *J. King Saud Univ – Comp Inform Sci* 2022;**34**:4514–4523.

Chen T, Guestrin C. XGBoost: A scalable tree boosting system. Procs. 22nd ACM SIGKDD Int. Conf. on Knowledge Discovery and Data Mining, KDD ’16, ACM. 2016:785–794 https://doi.org/10.1145/2939672.2939785.

Chowdhury S et al. Global health burden of ambient $PM_{2.5}$ and the contribution of anthropogenic black carbon and organic aerosols. *Environ Int* 2022;**159**:107020.

Crippa M et al. High-resolution temporal profiles in the Emissions Database for Global Atmospheric Research. *Sci Data* 2020;**7**:121.

Denier van der Gon H et al. Documentation of CAMS emission inventory products, 2023 https://doi.org/10.24380/Q2SI-TI6I; 2023.

Ehrhart S et al. Two new submodels for the Modular Earth Submodel System (MESSy): New Aerosol Nucleation (NAN) and small ions (IONS) version 1.0. *Geosci Model Dev* 2028;**11**:4987–5001.

Fountoukis C, Nenes A. ISORROPIA II: a computationally efficient thermodynamic equilibrium model for K^+–Ca^{2+}–Mg^{2+}–NH_4^+ –Na^+–SO_4^{2-}–NO_3^- –Cl^-–H_2O aerosols. *Atmos Chem Phys* 2007;**7**:4639–4659.

Georgiades P et al. Global high-resolution ultrafine particle number concentrations through data fusion with machine learning. *Nat Sci Data* 2025;**12**:1790.

GBD 2019 Risk Factors Collaborators. Global burden of 87 risk factors in 204 countries and territories, 1990–2019: a systematic analysis for the Global Burden of Disease Study 2019. *Lancet* 2020;**396**:1223–1249.

Guenther AB et al. The Model of Emissions of Gases and Aerosols from Nature version 2.1 (MEGAN2.1): an extended and updated framework for modeling biogenic emissions. *Geosci Model Dev* 2012;**5**:1471–1492.

Hersbach H et al. The ERA5 global reanalysis. *Q J Roy Meteorol Soc* 2020;**146**:1999–2049.

Hussein T, Sogacheva L, Petäjä T. Accumulation and coarse modes particle concentrations during dew formation and precipitation. *Aerosol Air Qual Res* 2018;**18**:2929–2938.

Jöckel P et al. Earth System Chemistry integrated Modelling (ESCiMo) with the Modular Earth Submodel System (MESSy) version 2.51. *Geosci Model Dev* 2016;**9**:1153–1200.

Johansson U, Boström H, Löfström T, Linusson H. Regression conformal prediction with random forests. *Machine Learn* 2014:**97**:155–176.

Jones RR et al. Land use regression models for ultrafine particles, fine particles, and black carbon in Southern California. *Sci Total Environ* 2020;**699**:134234.

Kaur J, Jhamaria C, Tiwari S, Singh Bisht D. Seasonal variation of ultrafine particulate matter (PM1) and its correlation with meteorological factors and planetary boundary layer in a semi-arid region. *Nat Environ Poll Technol* 2022;**21**:589–597.

Kerkweg A, Sander S, Tost H, Jöckel P. Technical note: Implementation of prescribed (OFFLEM), calculated (ONLEM), and pseudo-emissions (TNUDGE) of chemical species in the Modular Earth Submodel System (MESSy). *Atmos Chem Phys* 2006;**6**:3603–3609.

Kim B, Xu C, Barber RF. Predictive inference is free with the jackknife+-after-bootstrap. *Adv Neural Inform Proc Syst* 2020;**34** (NeurIPS 2020), https://proceedings.neurips.cc/paper/2020/.

Kirkby J et al. Ion-induced nucleation of pure biogenic particles. *Nature* 2016;**533**:521–526.

Kirkby J et al. Atmospheric new particle formation from the CERN CLOUD experiment. *Nat Geosci* 2023;**16**:948-957.

Klingmüller K et al. Revised mineral dust emissions in the atmospheric chemistry–climate model EMAC (MESSy 2.52 DU_Astitha1 KKDU2017 patch). *Geosci Model Dev* 2018;**11**:989–1008.

Kohl M et al. Numerical simulation and evaluation of global ultrafine particle concentrations at the Earth's surface. *Atmos Chem Phys* 2023;**23**:13191–13215.

Li Z et al. Aerosol and boundary-layer interactions and impact on air quality. *Nat Sci Rev* 2017;**4**:810–833.

Lloyd M, et al. Airborne nanoparticle concentrations are associated with increased mortality risk in Canada's two largest cities. *Am J Respir Crit Care Med* 2024;**210**:1338–1347.

Lundberg SM, Lee S-I. A unified approach to interpreting model predictions. *Adv Neural Inf Proc Syst* 2017;**30**, https://proceedings.neurips.cc/paper_files/paper/2017/.

Lundberg SM et al. From local explanations to global understanding with explainable AI for trees. *Nat Machine Intell* 2020;**2**:2522–5839.

McDuffie EE et al. A global anthropogenic emission inventory of atmospheric pollutants from sector- and fuel-specific sources (1970–2017): an application of the Community Emissions Data System (CEDS). *Earth Syst Sci Data* 2020;**12**:3413–3442.

Moore A, Bell M. XGBoost, A novel explainable AI technique. Prediction of myocardial infarction: A UK Biobank Cohort Study, Clinical Medicine Insights. *Cardiol*. 2022;**16**:117954682211.

Niu G et al. The variation in the particle number size distribution during the rainfall: wet scavenging and air mass changing. *Atmos Chem Phys* 2023;**23**:7521–7534.

Nohara Y, Matsumoto K, Soejima H, Nakashima N. Explanation of machine learning models using Shapley additive explanation and application for real data in hospital. *Comp Meth Progr Biomed* 2022; **214**: 106584.

Paasonen P et al. Continental anthropogenic primary particle number emissions. *Atmos Chem Phys* 2026;**16**:6823–6840.

Pesaresi M, Politis P. GHS-BUILT-V R2023A – GHS built-up volume grids derived from joint assessment of Sentinel2, Landsat, and global DEM data, multitemporal (1975-2030), https://doi.org/10.2905/AB2F107A-03CD-47A3-85E5-139D8EC63283 (2023).

Pezoa R, Salinas L, Torres C. Explainability of high-energy physics events classification using SHAP. *J Phys Conf Series* 2023;**2438**:012082, https://doi.org/10.1088/1742-6596/2438/1/012082.

Pond ZA, Saha PK, Coleman CJ, Presto AA, Robinson AL, Pope III CA. Mortality risk and long-term exposure to ultrafine particles and primary fine particle components in a national U.S. Cohort. *Environ Int* 2022;**167**:107439.

Pozzer A et al. Simulation of organics in the atmosphere: evaluation of EMACv2.54 with the Mainz Organic Mechanism (MOM) coupled to the ORACLE (v1.0) submodel. *Geosci Model Dev* 2022;**15**:2673–2710.

Pozzer A, et al. Mortality attributable to ambient air pollution: a review of global estimates. *GeoHealth* 2023;**7**:e000000.

Presto AA, Saha P, Robinson AL, Past, present, and future of ultrafine particle exposures in North America. *Atmos Environ* 2021;**10**:100109.

Pringle KJ. et al. Description and evaluation of GMXe: a new aerosol submodel for global simulations (v1). *Geosci Model Dev* 2010;**3**:391–412.

Riccobono F et al., Oxidation products of biogenic emissions contribute to nucleation of atmospheric particles. *Science* 2014;**344**:717–721.

Saha PK et al. High-spatial-resolution estimates of ultrafine particle concentrations across the continental United States. *Environ Sci Technol* 2021;**55**:10320–10331.

Sander R et al. The community atmospheric chemistry box model CAABA/MECCA-4.0. *Geosci Model Dev* 2019;**12**:1365–1385.

Schiavina M, Freire S, MacManus K. GHS-POP R2023A – GHS population grid multitemporal (1975-2030), https://doi.org/10.2905/2FF68A52-5B5B-4A22-8F40-C41DA8332CFE, 2023a.

Schiavina M, Melchiorri M, Pesaresi M. GHS-SMOD R2023A – GHS settlement layers, application of the Degree of Urbanisation methodology (stage I) to GHS-POP R2023A and GHS-BUILT-S R2023A, multitemporal (1975-2030), https://doi.org/10.2905/A0DF7A6F-49DE-46EA-9BDE-563437A6E2BA, 2023b.

Tost H, Jockel P, Kerkweg A, Sander R, Lelieveld J. Technical note: A new comprehensive SCAVenging submodel for global atmospheric chemistry modelling. *Atmos Chem Phys* 2006;**6**:565–574.

Trechera P et al. Phenomenology of ultrafine particle concentrations and size distribution across urban Europe. *Environ Int* 2023;**172**:107744.

Tsimpidi AP, Karydis VA, Pozzer A, Pandis SN, Lelieveld J. ORACLE: a module for the description of ORganic Aerosol Composition and Evolution in the atmosphere. *Geosci Model Dev* 2014;**7**:3153–3172.

van Donkelaar A et al. Monthly global estimates of fine particulate matter and their uncertainty. *Environ Sci Technol* 2012;**55**:15287–15300.

Wang Q et al. Traffic, marine ships and nucleation as the main sources of ultrafine particles in suburban Shanghai, China. *Environ Sci Atmos* 2023;**3**:1805–1819.

Zhao H, He Y, Shen J. Effects of temperature on electrostatic precipitators of fine particles and SO_3. *Aerosol Air Qual Res* 2018;**18**:2906–2911.
